# Identifying High Confidence microRNAs in the Developing Seeds of *Jatropha curcas*

**DOI:** 10.1038/s41598-019-41189-y

**Published:** 2019-03-14

**Authors:** Mingfeng Yang, Heshu Lu, Feiyan Xue, Lanqing Ma

**Affiliations:** Key Laboratory of Urban Agriculture (North China) Ministry of Agriculture, Beijing University of Agriculture, Beijing, 102206 China

## Abstract

MicroRNAs (miRNAs) are endogenously short noncoding regulatory RNAs implicated in plant development and physiology. Nine small RNA (sRNA) libraries from three typical seed developmental stages (young, intermediate, and mature) were generated by deep sequencing to identify the miRNAs of *J. curcas*, a potential oilseed crop for the production of renewable oil. Strict criteria were adopted to identify 93 high confidence miRNAs including 48 conserved miRNAs and 45 novel miRNAs. Target genes of these miRNAs were involved in a broad range of physiological functions, including gene expression regulation, primary & secondary metabolism, growth & development, signal transduction, and stress response. About one third (29 out of 93) miRNAs showed significant changes in expression levels during the seed developmental process, indicating that the miRNAs might regulate its targets by their changes of transcription levels in seed development. However, most miRNAs were found differentially expressed in the late stage of seed development, suggesting that miRNAs play more important roles in the stage when seed accumulating organic matters and suffering dehydration stress. This study presents the first large scale identification of high confidence miRNAs in the developing seeds of *J. curcas*.

## Introduction

Huge consumption of fossil fuel leading to higher and higher levels of carbon dioxide released into the atmosphere is a key concern in the world. Biodiesel, as an alternative for fossil fuel, is an environmentally friendly biofuel. *Jatropha curcas*, a perennial, drought-resistant small tree belonging to the family of Euphorbiaceae, can be grown on non-agricultural land without sacrificing agricultural land. Its seed has a high content of oil that can be reformed as biodiesel for diesel engines^1–3^. However, improving the seed yield, the oil content, and the oil quality remains challenging, and these problems prevents fulfilling the promise of this energy crop^4–6^. Understanding the molecular processes of seed development and oil metabolism is crucial to solve these problems. Much efforts have therefore been made to analyze the gene expression in all kinds of tissues in *Jatropha*, especially in the developing seeds^7–10^. The genomic sequences and protein-encoding genes obtained through genome analysis have laid the foundation for further exploration of the genetics and genomics of *J. curcas*^11–13^.

MicroRNAs (miRNAs) are endogenous small non-coding RNAs that act as post-transcriptional regulators of gene expression in animals and plants by targeting mRNAs for cleavage or translational repression^14–16^. In plants, miRNAs are encoded in intergenic regions, where they are typically transcribed by RNA Polymerase II as long polyadenylated transcripts called pri-miRNAs. Then pri-miRNAs are recognized and processed by DICER-Like1 (DCL1) into miRNA precursors (pre-miRNAs), which are further processed to generate 18~24 nucleotide (nt) mature miRNAs^16–18^. The mature miRNAs bind to target mRNAs for cleavage or translational inhibition, depending on the degree of complementarity between the miRNAs and its target mRNAs^15,18^. In recent years, researchers found that miRNAs play critical roles in plant development and growth as well as a range of physiological processes, including abiotic and biotic stress responses and probably lipid metabolism^19–21^.

To our knowledge, only several recent studies focused on the identification of miRNAs from *J. curcas*. Wang *et al*. cloned 52 putative miRNAs from leaves and seeds without providing information on the miRNA precursor sequence^22^. Vishwakarma and Jadeja used known plant miRNAs to search for *J. curcas* miRNAs from EST and GSS sequences, and only 24 putative conserved miRNAs were identified^23^. Galli *et al*. investigated miRNAs through small RNAs (sRNA) deep sequencing from mature seeds, and revealed 180 conserved miRNAs and 41 precursors as well as 16 novel pre-miRNAs, but miRNAs involved in other developmental stages are yet to be found^24^. Furthermore, quite a few miRNAs identified in these reports could not be qualified as high confidence miRNAs according to the current strict criteria^25,26^.

In this study, high confidence miRNAs from *J. curcas* were identified through the deep sequencing of sRNA from three typical developing stages of seeds (young, intermediate and mature). The prediction of miRNA targets indicates that these miRNAs are involved in a wide range of physiological processes. In addition, differentially expressed miRNAs were found mainly in the late stages, providing important information on the possible function of miRNAs in seed development.

## Results and Discussion

### Deep Sequencing of sRNAs

To identify miRNAs and their expression patterns in the developing seeds of *J. curcas*, nine sRNA libraries from three typical developmental stages of seeds (young, intermediate and mature seeds; Supplementary Fig. S1) with three biological replicates were generated and sequenced by next-generation sequencing (NGS), resulting in 16.7~21.6 million raw reads for each sample and 162.7 million raw reads in total, which was a deep resource for extensive discovery of miRNAs. After trimming the adaptors, filtering out the low quality reads and noise, we obtained 13.8~18.9 million clean reads for each of the nine sRNA libraries, and no less than 95% of the reads had a high Detection Accuracy (>99.9%) (Table 1).

**Table 1:** Deep sequencing of sRNAs from 9 libraries of *J. curcas* developing seeds.

Samples	BMK-ID	Raw reads	Low quality reads	Containing ‘N’ reads	Length < 18	Length > 30	Clean reads	Q30(%)	Clean/Raw (%)
Mature1	S01	19043797	0	5092	1233213	1949732	15855760	95.0	83.3
Mature2	S02	18295787	0	5067	1298051	1603578	15389091	95.2	84.1
Mature3	S03	17673444	0	4844	1277677	1293943	15096980	95.3	85.4
Intermediate1	S04	17064128	0	4787	1551724	1000675	14506942	95.2	85.0
Intermediate2	S05	16791723	0	4648	1975254	1061054	13750767	94.9	81.9
Intermediate3	S06	17893351	0	4814	1099554	1248391	15540592	95.2	86.9
Young1	S07	21600131	0	16164	1767028	959579	18857360	95.0	87.3
Young2	S08	16697519	0	12765	1645390	745144	14294220	95.2	85.6
Young3	S09	17647543	0	13223	512391	1686789	15435140	95.1	87.5

Samples: samples named by authors; BMK-ID: samples named by Biomarker Technologies; Raw reads: raw data of sequencing; Low quality reads: reads with >20% bases <Q30, where Q30 mens one error in 1000 bases; Containing ‘N’ reads: unknown bases >10%; Length <18nt reads: less than 18 nucleartides after trimming adaptors; Length > 30nt reads: more than 30 nucleartides after trimming adaptors; Clean reads: remaining reads after filtering; Q30 (%): percentage of reads with quality value > = Q30 (Q30 means Detection Accuracy >99.9%). Clean/Raw (%): percentage of Cean reads in Raw reads.

The clean reads were mapped to the SILVA rRNA, Rfam, tRNAdb, and Repbase databases to annotate the composition of the sRNA population. It showed that rRNA had the highest read frequency (19.65~41.56%) followed by tRNA (1.46~3.52%) in the total clean reads of every library. Repetitive elements, scRNA, snRNAs and snoRNA were less frequent in the sRNA library. Taken together, 55.24~77.1% of the clean reads were unannotated reads for the nine sRNA libraries, and these unannotated reads could be used for miRNA detection (Table 2).

**Table 2:** Small RNA types in 9 sRNA libraries of *J. curcas* developing seeds.

Samples	S01		S02		S03		S04		S05		S06		S07		S08		S09	
Type	Number	%	Number	%	Number	%	Number	%	Number	%	Number	%	Number	%	Number	%	Number	%
Total	15855760		15389091		15096980		14506942		13750767		15540592		18857360		14294220		15435140	
rRNA	3459293	21.8	4660796	30.3	2966676	19.7	4877224	33.6	5714339	41.6	4746557	30.5	7456192	39.5	5159632	36.1	4192866	27.2
scRNA	0	0.0	0	0.0	0	0.0	0	0.0	0	0.0	0	0.0	0	0.0	0	0.0	0	0.0
snRNA	0	0.0	0	0.0	0	0.0	0	0.0	0	0.0	0	0.0	0	0.0	0	0.0	1	0.0
snoRNA	11202	0.1	9190	0.1	9954	0.1	13000	0.1	12554	0.1	14243	0.1	16924	0.1	14447	0.1	10727	0.1
tRNA	452768	2.9	541892	3.5	444180	2.9	316861	2.2	405727	3.0	325241	2.1	448510	2.4	343103	2.4	224897	1.5
Repbase	18492	0.1	16968	0.1	36403	0.2	16925	0.1	22427	0.2	16449	0.1	24813	0.1	22772	0.2	10357	0.1
Unannotated	11914005	75.1	10160245	66.0	11639767	77.1	9282932	64.0	7595720	55.2	10438102	67.2	10910921	57.9	8754266	61.2	10996292	71.2

Types: small RNA types; Total: Total clean reads; rRNA: ribosomal RNA; scRNA: small cytoplasmic RNA; snRNA: Small nuclear RNA; snoRNA: small nucleolar RNA; tRNA: Transfer RNA; Repbase: repetitive elements; Unannotated: The remaining reads without rRNAs, scRNA, snRNAs, snoRNAs, tRNA, or repetitive elements; %: the percentage of small RNAs in total clean reads.

Among the clean reads, the most abundant sequences were 21 nt (8.0%), 22 nt (6.1%), 23 nt (10.5%), and 24 nt (45.4%) (Supplementary Table S1). This distribution pattern of the sRNA size was similar to those seeds from other species, such as the developing seeds of canola^27,28^ and safflower^29^, and the mature seeds of *J. curcas*^24^. In these reports, the most abundant sRNA types are 21~24-nt long and 24-nt sRNA is also the most abundant one (>40%), indicating that 21~24-nt long sRNA might be the main sRNA products, and these plants might have similar sRNA biogenesis patterns in seeds.

### Identification of High Confidence miRNAs

To analyze the unannotated reads for discovery of miRNAs, the miRDeep2 core algorithm with modifications for plant miRNAs was employed^30,31^. In brief, the unannotated reads were mapped onto the genome of *J. curcas*, and the flanking sequences of the mapped sites were extracted as candidate miRNA precursors. The mfe (minimal free energy) of the precursor structure, read counts of mature and star miRNA sequences, randfold *p*-values, and minimum 60% nucleotides paired in the candidate mature and star miRNAs, were considered and scored by the miRDeep2 core algorithms. In total, 108 potential miRNAs were identified in the developing seeds of *J. curcas*.

The stem-loop structures and other characters of miRNA precursors were displayed in miRDeep2 outputs (Supplementary Fig. S2 and Supplementary Table S2). These data were further manually examined for mismatches between the miRNA and star miRNA, the number of asymmetric bulges in the stem region, the size of asymmetric bulges, 3′ overhangs in duplex, dominance of the miRNA relative to other sRNAs in abundance, and precise miRNA/star miRNA excision, to annotate high confidence microRNAs according to the rules detailed by Meyers *et al*.^25,32^. Four rules must be followed if miRNAs could be accepted as high confidence ones: (1) The miRNA and star miRNA form a duplex with two nt 3′ overhangs (if star miRNA is absent, candidate miRNA must be found in multiple, independent libraries); (2) four or fewer mismatches between the miRNA and the other arm of the hairpin; (3) not more than one asymmetric bulge and less than two bases in the bulge, especially within the duplex; and (4) stem-loops that slightly violate one of these criteria could still be annotated as miRNAs, if precise miRNA/star miRNA excision is shown, i.e., more than 75% sRNA localized in one arm of miRNA/star miRNA duplex^25^. According to these rules, 93 high confidence miRNAs were obtained from the developing seeds (Supplementary Fig. S2 and Supplementary Table S2). That is to say, about 86% (93/108) miRNAs were qualified as high confidence miRNAs, confirming the efficiency and accuracy of the modified miRDeep2 in plant miRNA identification^30,31^. Most of the unqualified miRNAs were ruled out due to the imprecise miRNA/star miRNA excision or improper 3′ overhangs. If more stringent filtering strategies were adopted, three more rules should be added according to Kozomara and Griffiths-Jones^26^: (5) At least 10 reads must map with no mismatches to each of the two possible mature microRNAs derived from the hairpin precursor; (6) at least 50% of reads mapping to each arm of the hairpin precursor must have the same 5′ end; and (7) the predicted hairpin structure must have a folding free energy of <−0.2 kcal/mol/nt. Finally, we got 45 very high confidence miRNAs satisfying all the seven rules mention above (Supplementary Table S2). Almost all the 48 miRNAs failed to be qualified for very high confidence ones due to failure to satisfy the fifth rule: less than 10 reads of star miRNAs mapped with no mismatches. Considering the fact that star miRNAs are expressed at low level or even hardly detected for some miRNAs, some miRNAs could be approved as high confidence ones if they meet all the ancillary criteria^25^. For example, in the absence of star miRNA confirmation, a clear dominance of the miRNA sequences from one arm of a predicted stem-loop was found (Supplementary Fig. S2). Furthermore, the annotation was well supported by miRNA identification from multiple, independent libraries. Most of the 93 miRNAs were found in 9 independent libraries, except that Jcr4S02434_14511 and Jcr4S00022_291 could not be detected in young seeds. All the miRNAs identified in this work had high read counts. For instance, Jcr4S00096_1114 had the smallest (285) and Jcr4S01201_8767 had the highest read counts (741967). These features could be considered as ancillary criteria that strongly bolster miRNA annotations as suggested by Meyers *et al*.^25^ When we checked the structures of *J. curcas* miRNAs published in previous reports by Wang *et al*.^22^ and Galli *et al*.^24^, less than half of the miRNAs could satisfy Meyer’s rules (2) and (3) mentioned above, let alone other rules. Therefore, the miRNA screening criteria adopted by this work are stringent enough to qualify the 93 miRNAs as high confidence miRNAs.

**Figure 1:**
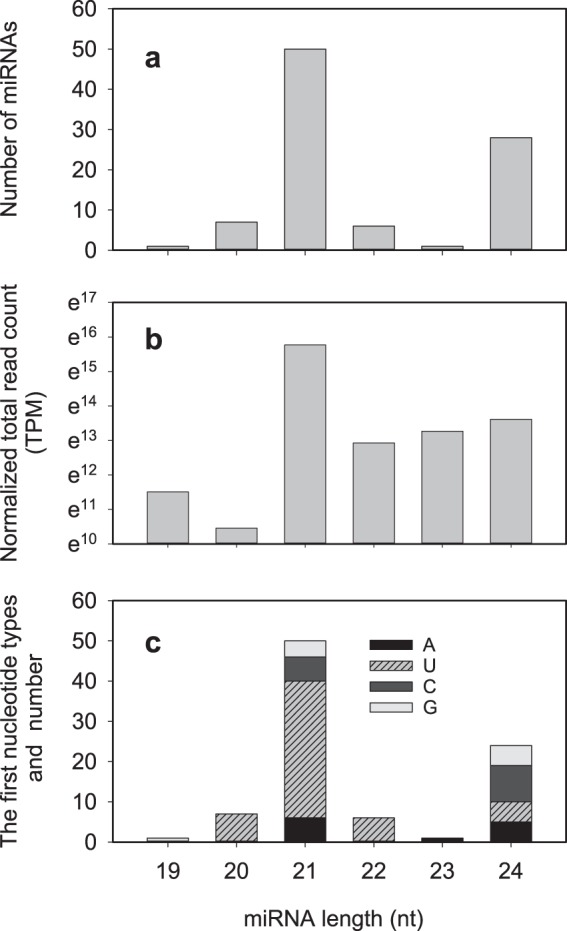
Characterization of identified miRNAs in *J. curcas* developing seeds. (**a**) The number of miRNAs along with the mature miRNA length. (**b**) The distribution of reads along with the mature miRNA length. (**c**) The presence of the first nucleotide of miRNAs along with the mature miRNA length.

The lengths of the 93 mature miRNAs varied from 19 nt to 24 nt. More than half (50/93) of miRNAs were 21 nt, followed by 24-nt miRNAs (28) (Fig. 1a). Accordingly, 21-nt and 24-nt sRNAs were the most abundant ones (Fig. 1b). Plants have at least four Dicer-like proteins (DCL1-4), among which DCL1 mainly produces 18~21-nt long sRNA while DCL2, DCL3, and DCL4 produce 22-, 24-, and 21-nt long sRNA, respectively, and 21, 22, and 24 nt are typical sizes for plant Dicer-like (DCL)-derived products^16^. Although 24-nt sRNAs accounted for almost half (45.4%) of the total sRNAs and 21-nt sRNAs took up much less (8.0%) (Supplementary Table S1), it is interesting to note that more than half miRNAs (50/93) were 21 nt in length, which is quite accordant to the fact that DCL1 is responsible for most plant miRNA biogenesis and plant miRNAs are predominately 21 nt in length^18^. However, both the 24-nt miRNAs and 24-nt sRNAs were the second large population in total clean reads, indicating DCL3 might play a part in the sRNA production in the developing seeds. We also determined the frequency of the first base of mature miRNAs, and it showed that the 20-, 21-, and 22-nt miRNAs preferentially started with ‘U’ (7/7, 34/50, and 6/6, respectively), while 24-nt miRNAs preferred ‘C’ at the first base (9/24) (Fig. 1c). Similar base bias trend for 20-, 21-, and 22-nt miRNAs is also found in other plant species, such as Camellia^33^ and canola^28^. This is well in accordance to that plant miRNAs prefer to begin with a ‘U’ residue^34^. However, the first base of 24-nt miRNAs prefer ‘A’ in Camellia^33^ and canola^28^. The first base bias may determine their assembling into different AGO complexes and might result in a different regulatory outcome in the developing seeds of *J. curcas*^16^.

### Identification of Conserved and Novel miRNAs

High confidence miRNAs were then aligned to known miRNAs from miRBase. A total of 48 miRNAs shared homology with known miRNAs were identified as conserved miRNAs, and 45 miRNAs without homology, novel miRNAs (Supplementary Table S2). The 48 conserved miRNAs belonging to 23 families (Jcu-MIR156, 159, 160, 162, 164, 166, 167, 168, 171, 172, 319, 390, 393, 394, 395, 396, 398, 403, 408, 477, 827, 1446, and 6445) could be found in at least one plant species except for *J. curcas*, and most (39/48) were identified in more than 10 species (Supplementary Table S2). Jcu-MIR156 and MIR166 were the biggest families with 6 members each, followed by Jcu-MIR319 (5 members). Four families (Jcu-MIR156, 159, 390, and 6445 (named nMIR001-5p in Galli’s report)) were also detected in the mature seeds of *J. curcas* in a previous report^24^. Jcu-MIR166 and Jcu-MIR167 families were both discovered in mature seeds based on deep sequencing^24^ and bioinformatic prediction based on expressed sequence tags^23^. Jcu-MIR167 (JcumiR027 in Wang’s work) was also identified in the leaf of *J. curcas* by cDNA cloning method, but not in the seed tissues^22^. The 45 novel miRNAs belonged to 34 families (named Jcu-nMIR001~Jcu-nMIR034). In previous reports, Jcu-nMIR020 (nMIR002-5p in Galli’s work) was only observed in the mature seeds^24^ and Jcu-nMIR005 (JcumiR019-3p in Wang’s work) was identified in the leaves instead of seed tissues of *Jatropha*^22^. However, Jcu-MIR167 and Jcu-nMIR005 were detected in all the three developing seed stages with extremely high read counts (>400,000) in this work, but neither were detected in the seeds of *Jatropha* by cDNA cloning method^22^, implying that even high abundance of miRNAs could not guarantee their detectability by the cloning method, because some miRNAs could be missed by chance when cloned into the pGEMT vector and sequenced^22^. To our knowledge, a total of 17 conserved (except Jcu-MIR156, 159, 166, 167, 390, and 6445) and 32 novel (except Jcu-nMIR020 and Jcu-nMIR005) miRNAs families were identified in *J. curcas* for the first time.

### Prediction of miRNA Targets

In plants, as a post-transcriptional regulator, the mature miRNA binds to target mRNAs by their complementarity for cleavage or translational repression, and therefore miRNA target prediction is crucial for understanding the functions of miRNAs^14–16,18^. Based on the complementarity, target mRNAs of *J. curcas* miRNAs were predicted by using the web-based psRNATarget^35^ and Targetfinder software^36^.

A total of 331 potential target genes were found for 73 miRNAs with an average of 4.5 targets per miRNA (Supplementary Table S2). Some miRNAs had a larger number of targets, such as Jcu-MIR156 and Jcu-MIR396 (>12 targets for each family members), which were also observed to have the most targets in cassava^37^; The target genes of 22 conserved and 24 novel miRNA families were obtained. In other words, targets were spotted for almost all conserved miRNAs (46/48) and conserved miRNA families (22/23), whereas only for about half of novel miRNAs (27/45) and novel miRNA families (24/34), and a similar trend was observed in the targets determination in a previous report^24^. These results are in line with the idea that novel plant miRNAs, in contrast to deeply conserved miRNAs, are more divergent and tend to lack targets^38^.

A total of 188 unique target sequences were obtained after removing the redundancy in the 331 potential targets for the 73 miRNAs, and the functions of the 188 unique targets were annotated according to previous reports^11,12^ or by using BLASTX against NCBI nr and Swiss-Prot/Uniprot protein databases (Supplementary Table S3), followed by a GO analysis to evaluate their putative functions in regards to the cellular component, the molecular function and the biological process (Supplementary Fig. S3). The majority of miRNA targets are localized in nucleus or membrane, and some are in cytoplasm, ribosome, chloroplast, mitochondrion, cell wall, or even extracellular region; most miRNA targets participate in DNA binding, ATP binding, or had transcription factor activity; the miRNA targets mainly participate in DNA-templated regulation of transcription, oxidation reduction, developmental process, metabolic processes, protein phosphorylation or ubiquitination, electron transport, and signaling pathways (Supplementary Fig. S3). Most targets are involved in transcription regulation, and have a broad range of physiological functions. The function categories of miRNA targets in GO analysis are overlapped and complicated, and we therefore categorized the targets roughly into five aspects of plant physiological process according to the annotation in NCBI nr, Uniprot data, and previous reports: gene expression regulation, primary & secondary metabolism, growth & development, signal transduction, and stress response. To be concise, the miRNAs identified in this work are parenthesized behind their targets in the following text.

The first aspect comprises genes involved in all levels of gene expression regulation: DNA replication, transcription, post-transcriptional regulation, translational process and posttranslational modification. Non-structural maintenance of chromosomes element 4 (Jcu-MIR6445) is involved in DNA replication, recombination and repair. Most targets are involved in gene transcription process and some are well known families of transcription factors, such as DELLA protein, leucine zipper protein, AP2/ERF and B3 domain-containing protein, NAC domain-containing protein, WRKY, squamosa promoter-binding-like protein, MYB, GAMYB, etc (Supplementary Table S3). The targets are also involved in post-transcriptional regulation, such as endoribonuclease Dicer homolog 1 (Jcu-MIR162) and argonaute 2 (Jcu-MIR403), which contribute to the production of miRNA or siRNA. Pentatricopeptide repeat (PPR) proteins (Jcu-nMIR021) are a large family of modular RNA-binding proteins which mediate several aspects of gene expression primarily in organelles but also in nucleus. These proteins facilitate processing, splicing, editing, stability and translation of RNAs^39^, and thus it is classified as RNA processing and modification. Translation initiation factor eIF-2B subunit (Jcu-nMIR025) and eukaryotic peptide chain release factor GTP-binding subunit ERF3A (Jcu-MIR1446) are typical factors involved in translational process. Some target genes function at posttranslational modification level, in which protein turnover is regulated through ubiquitin-dependent pathway, such as RING-H2 finger protein ATL5 (Jcu-nMIR011), F-box only protein 6 (Jcu-MIR394), E3 ubiquitin-protein ligase (Jcu-MIR396), ubiquitin-conjugating enzyme (Jcu-nMIR021), and ubiquitin-like modifier-activating enzyme (Jcu-MIR172). The results indicate that miRNAs are involved indirectly in all levels of gene expression regulation, though miRNAs per se are post-transcriptional regulators.

The second aspect includes the genes involved in primary & secondary metabolism. Some target genes play roles in primary metabolism, i.e., protein, carbohydrate, and lipid metabolism. A sec. 1 family domain-containing protein MIP3 (Jcu-nMIR003) is required for proper maturation of seed storage proteins and it forms a complex with MAG2, ZW10/MIP1 and MIP2 on the endoplasmic reticulum which may be responsible for efficient transport of seed storage proteins. The 1, 4-alpha-glucan-branching enzyme 2–2 (Jcu-nMIR011) is involved in starch biosynthetic process. Eight miRNA targets are related to lipid metabolic pathways (Supplementary Table S3), among which three targets were involved in the lipid or fatty acid biosynthetic processes: cytochrome P450 (Jcu-MIR156f) catalyzes the omega-hydroxylation of various fatty acids, which is important for cutin biosynthesis, trichome differentiation, establishment of apical dominance and senescence^40^; GPI mannosyltransferase 1 (Jcu-MIR403) works in the pathway of cosylphosphatidylinositol-anchor biosynthesis, a part of glycolipid biosynthesis; and malonate-CoA ligase (Jcu-MIR403) catalyzes the formation of malonyl-CoA directly from malonate and CoA. Among the eight targets related to lipid metabolic pathways, five targets play a part in the lipid catabolic processes: monoacylglycerol lipase ABHD6 (Jcu-nMIR013), sn1-specific diacylglycerol lipase beta (Jcu-nMIR008), and phospholipase D zeta 1(Jcu-MIR827) are lipase; long-chain-alcohol oxidase FAO2 (Jcu-MIR1446) is involved in the omega-oxidation pathway of lipid degradation; acyl-coenzyme A thioesterase 9 (Jcu-nMIR011), catalyzes the hydrolysis of acyl-CoAs to the free fatty acids and CoAs, probably regulating intracellular levels of acyl-CoAs, free fatty acids and CoASH. Although increasing the oil content and improving the oil quality are still a challenging task^5,41^, the discovery of these miRNAs involved in lipid metabolism might provide an alternative way to solve the problems; for example, Jcu-nmiR011 might have a potential application in controlling the level of free fatty acids in the seed oil of *J. curcas*. Except for the primary metabolism, target genes involved in the secondary metabolism were also identified: deacetylvindoline O-acetyltransferase (Jcu-MIR156f) was shown to be involved in the biosynthesis of vindoline, a precursor of vinblastine and vincristine^42^; premnaspirodiene oxygenase (Jcu-MIR159) functions in the biosynthesis of solavetivone, a potent antifungal phytoalexinin^43^; chalcone synthase 2 (Jcu-nmiR014) participates in the biosynthesis of flavonoid, which is an important secondary metabolite in plants; and laccase-9 (Jcu-MIR398) is an important enzyme in lignin degradation and detoxification of lignin-derived products, which has become a focus recently for environmental protection. These results suggest that miRNAs play important roles in a wide range of plant primary and secondary metabolism and have promising application in agriculture, medicine and environment protection.

The third aspect is growth & development, including genes involved in cell division, cell differentiation and seed development. Growth-regulating factors (GRF1, 2, 4, 5, 7, 8 and 9) targeted by Jcu-MIR396 are transcription activators associated with the cell expansion of leaves and cotyledons. The evidences obtained from Arabidopsis, rice, and maize proved that miR396-GRF, a conserved regulatory network in plants, plays an important role in leaf growth and seed development, acting in the regulation of meristem function, at least partially through cell proliferation control^44,45^. Protein CUP-SHAPED COTYLEDON 2 (Jcu-MIR164) involved in meristem initiation and cell division patterns is also a target of Ath-MIR164 in Arabidopsis^46^. Coiled-coil domain-containing protein SCD2, AUGMIN subunit 5, and trafficking protein particle complex II-specific subunit targeted by Jcu-MIR156, 477, and nmiR001 respectively, take part in cell division and cell expansion^47–49^. Callose synthase 9 (Jcu-MIR319), cellulose synthase-like protein G3 (Jcu-MIR395), and leucine-rich repeat extensin-like protein 2 (Jcu-nMIR005) are involved in cell wall organization. Homeobox-leucine zipper protein ATHB-8, ATHB-14 (PHABULOSA), ATHB-15 (CORONA), and REVOLUTA belong to class III homeodomain-leucine zipper genes, which are transcription factors playing overlapping and different roles in the cell differentiation, vascular tissue formation, integument development, and embryogenesis^50–52^. Previous reports showed that ATHB15 is targeted by MIR166 in Arabidopsis^53^. Our results indicate that class III homeodomain-leucine zipper genes with related functions are all targeted by Jcu-MIR166, which might be an efficient strategy for the regulation of target genes coordinately. Transcription factor TCP family (2, 3 and 4), targeted by Jcu-MIR319, plays a pivotal role in cell division and leaf development and is required during early steps of embryogenesis^54,55^. As MIR319-TCP regulates leaf development in Arabidopsis^55^, MIR319-TCP pathway is probably conserved and might be related to seed development in *J. curcas*. Floral homoerotic protein APETALA 2 (Jcu-MIR172) activity is required in plant ovule development and seed development in addition to its known function in floral development^56^. It is worth to mention that miRNA-target network must be complicated in controlling plant development; for instance, MIR319 targets transcription factor TCP, negatively regulating the expression of CUP-SHAPED COTYLEDON through the induction of MIR164^57^.

The fourth aspect is signal transduction, including genes targeted by Jcu-MIR156, 159, 390, 393, 394, 396, 403, 827, nMIR010, nMIR014, nMIR021, nMIR024, and nMIR033. Some targets are under the control of plant hormones. Histidine kinase 3 (Jcu-nMIR033a) may participate in cytokinin-activated signaling pathway that regulates many developmental processes including seed germination, cell division, and seed size^58^. Auxin signaling F-box 2 (Jcu-MIR393) is involved in embryogenesis regulation by auxin, and it is shown to be targeted by MIR393 for embryogenesis in Arabidopsis^59^. Auxin response factor 16 (Jcu-MIR160), though classified as transcriptional factors, belongs to auxin-activated signaling pathway^60^. These genes may play a role in cell division and cell differentiation, and thus eventually in seed growth and formation under the control of hormones and miRNAs. Calmodulin is known to be involved in signal transduction by mediates the control of a large number of enzymes, ion channels and other proteins. Calmodulin-7 (Jcu-nMIR021) is transcription factor in Arabidopsis seedling development^61^. These results suggest that miRNAs should control a large range of plant activities since some signal transduction pathways are under the regulation of miRNAs.

Finally, it is no wonder that a lot of targets are involved in stress response, since seeds should be suffered desiccation and other stress during developmental process. Late embryogenesis abundant protein (Jcu-MIR394) is a group of well known proteins which may play an essential role in seed survival and in controlling water exchanges during seed desiccation. At least four targets of miR159 are related to defense and stress response, such as superoxide dismutase and peroxidase (Supplementary Table S3). AP2/ERF and B3 domain-containing transcription factor (Jcu-MIR156) may be involved in the regulation of gene expression by stress factors and by components of stress signal transduction pathways according to Uniprot annotation. In Arabidopsis, MIR156, MIR159 and MIR394 are known as dehydration stress-responsive miRNAs. DEAD-box ATP-dependent RNA helicase 38 (Jcu-MIR393) is essential for mRNA export from the nucleus and plays an important role in the positive regulation of CBF/DREB transcription factors^62,63^, and *J. curcas* DREB gene expression is induced by cold, salt and drought stresses^64^.

### Differentially Expressed miRNAs During Seed Development

**Figure 2:**
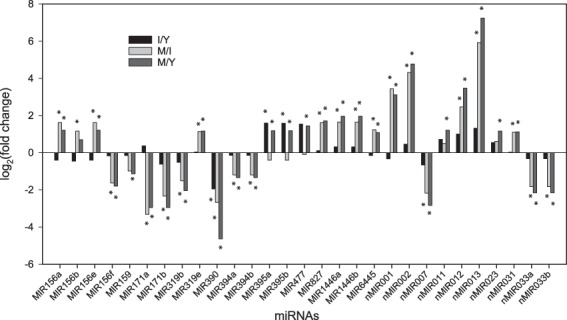
The differentially expressed miRNAs during seed development of *J. curcas*. The miRNA expression levels were estimated for each sample by mapping sRNA back onto the miRNA precursor sequence, and read count for each miRNA was obtained from the mapping results. Differential expression analyses of miRNAs were performed based on Read counts using the DESeq. 2, in which *p* values were adjusted using the Benjamini and Hochberg’s approach for controlling the false discovery rate (FDR). The variation of miRNA levels in three different developmental stages, i.e., young (Y), intermediate (I) and mature (M) seeds were shown as the base-2 logarithm of ratios (log_2_ fold change) between different developmental stages (I/Y, M/I and M/Y). The miRNAs whose expression levels between any two of the three different developmental stages changed significantly (|log_2_ fold change| > 1 and FDR adjusted *p* < 0.05) were assigned as differentially expressed miRNAs (indicated by *).

Plant miRNAs play pivotal roles in the regulation of plant growth and development and therefore it is crucial to outline the spatiotemporal expression patterns of miRNAs. To gain insight into the possible developmental stage-dependent roles of miRNAs in *Jatropha*, the expression levels and patterns of miRNAs in seed were determined at three different developmental stages. The miRNA expression levels were shown as TPM values which were comparable between different samples^36^. About one third (29/93) were differentially expressed miRNAs (fold change >2, and *p* < 0.05) (Supplementary Table S4), which were presented as the base-2 logarithm of fold change of expression levels between young, intermediate, and mature stages (Fig. 2). In the 29 differentially expressed miRNAs, 11 miRNAs were down-regulated whereas 18 miRNAs were up-regulated, and the variation was continuous from young through mature stages without fluctuation (Fig. 2). Among these differentially expressed miRNAs, Jcu-MIR171a/b and Jcu-MIR390 were down-regulated most significantly. Jcu-MIR171b targets scarecrow-like protein 15 (XM_012223680.2), which is a transcription factor involved in plant development. MIR390 targets protein NRT1/ PTR FAMILY 4.6 (XM_012224813.2) which is involved in cellular abscisic acid (ABA) transport and uptake^65^. Jcu-nMIR001, nMIR002 and nMIR013 were very highly expressed in mature seeds, but hardly detected in young and intermediate stages (Supplementary Table S4). Chloroplastic photosynthetic NDH subunit of subcomplex B 3 (XM_012223240.2; target of Jcu-nMIR001) shuttles electrons in the photosynthetic electron transport in photosystem I to produce energy^66^. Another target of Jcu-nMIR001 is trafficking protein particle complex II-specific subunit 130 (XM_012215242.2), which is responsible for growing cell plate in mitotic active cells^48^. Jcu-nMIR002 targets auxin-responsive protein SAUR50-like (XM_020682576.1), which promotes cell expansion during plant growth^67^. Jcu-nMIR013 targets monoacylglycerol lipase ABHD6 (XM_012226208.2) which participates catabolic process of lipids, and the high level of Jcu-nMIR013 in mature seeds should repress the lipid catabolic process and so benefit the lipid accumulation. *Jatropha* 11 kD late embryogenesis abundant protein belongs to late embryogenesis abundant (LEA) group 1 (XM_012232372.2) involved in the acquisition of desiccation tolerance during late phase of embryogenesis^68,69^. It is targeted by Jcu-MIR394, which was down-regulated significantly during seed maturation, probably facilitating the accumulation of LEA protein at mature stage to improve desiccation tolerance. However, it is hard to find an unambiguous inverse corelation between the differentially expressed miRNAs and the target mRNAs levels (data not shown), because miRNAs might play a part role in the regulation of mRNA levels and some targets were regulated by miRNAs at translational level instead of cleavage. Degradome sequencing should be a better choice to solve this problem^28^.

**Figure 3:**
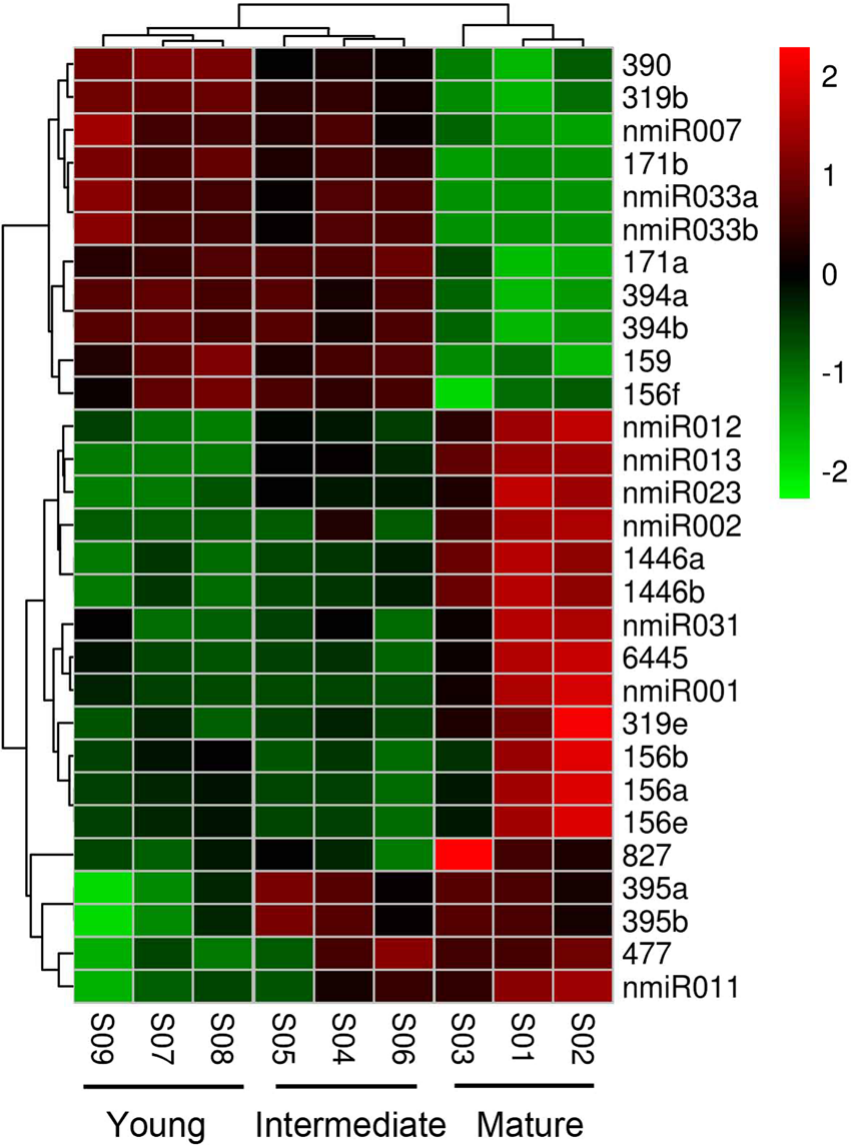
The global expression of the 29 differentially expressed miRNAs in three developmental stages (young, intermediate and mature) of *J. curcas* seeds. Three independent biological replicates of developing seeds at each stage (young: S07, S08 and S09; intermediate: S04, S05, and S06; mature: S01, S02, and S03) were shown. The colors indicate relative expression levels of miRNAs, where high levels are indicated by red, and low levels are green.

To our surprise, most differentially expressed miRNAs were observed during seed development from intermediate to mature stages, and only three miRNAs (Jcu-miR390 and Jcu-395a/b) were observed from young to intermediate. Reverse transcription (RT) quantitative real-time PCR (qPCR) was then performed for the validation of differentially expressed miRNAs. In addition to Jcu-miR390 and Jcu-395a/b, three more miRNAs (Jcu-miR477, Jcu-nMIR011, and Jcu-nMIR023) were observed to be differentially expressed from young to intermediate seeds, which was still much less than the number of differentially expressed miRNAs from young to mature (29 miRNAs). Nevertheless, the trend of expression variation of miRNAs from RT-qPCR agreed very well with the sequencing results in terms of up or down regulation (Supplementary Fig. S4). A heatmap was made to gain an overview of the differentially expressed miRNAs (Fig. 3). The global expressions of the 29 differentially expressed miRNAs had high correlations among three replicates, and samples from three different developmental stages of *J. curcas* seeds were grouped into three clades accordingly, suggesting that the data sets were reliable and ideal for statistical analysis for differentially expressed miRNAs (Fig. 3). The heatmap profiles were similar between young seeds and intermediate ones, which might be due to the young seeds were in the histodifferentiation stage and the intermediate seeds were in the early transition from histodifferentiation to seed filling stage^70^, and the two stages might have overlapped physiological process such as cell division and tissue differentiation. Nevertheless, a huge difference for the global profile of miRNA levels in mature seeds was observed when compared with the young and intermediate stages (Fig. 3). During the transition from intermediate to mature stage, seeds were developing much faster than before in regards to length, fresh weight and dry weight, and were suffering desiccation stress (Supplementary Fig. S1). These results from deep sequencing and RT-qPCR both suggested that the post-transcriptionally regulation of genes by miRNAs mainly happened at the late seed developmental stages when seeds experienced organic matter accumulation and desiccation stress.

### Identification of IsomiRNAs

Some miRNA variants, known as isomiRNAs (isomiRs), have additional nucleotides in the 5′ or 3′ terminus when compared to canonical miRNAs, which might be a consequence of inaccuracies in Dicer pre-miRNA processing or post-transcriptional modification^71–74^. A total of 36 isomiRs belonging to 13 families (Jcu-MIR 159, 166, 167, 319, 393, 396, nMIR003, nMIR004, nMIR005, nMIR010, nMIR022, nMIR026, nMIR027; see Supplementary Table S6) were detected. Among these isomiRs, thirty 3′ isomiRs (3′ deletion, extension and nontemplated modifications), seven 5′ isomiRs (5′ deletion and extension) and four polymorphic isomiRs (only harbor distinct nucleotide compositions within the miRNA sequences) were identified, which agrees to the fact that 3′ isomiRs are the most frequently observed type of isomiR in animals and plants, in terms of both number and abundance^71^. Interestingly, no polymorphism within the seed sequence (2–9 nt) was observed, and it might suggest that the modification in these miRNA variants has little effect on target selection, because most of the isomiRs had similar targets with the canonical miRNAs (data not shown). The target mRNAs are recognized by multiple isomiRs plus the canonical miRNA, which might increase the specificity and efficacy of the target silencing^75^.

When inspecting the expression of these isoform variants among nine libraries, most isoforms displayed a similar trend of expression during seed development, whereas only a few isoforms had significant divergence. It is noteworthy that isomiR319-1 increases while isomiR319-3 decreases sharply during the seed developing process (Supplementary Table S5), suggesting that isomiR members in the same family might have different regulation mechanism.

## Conclusions

The identification of 93 high confidence miRNAs and their targets provides useful insights into miRNA-mediated regulatory mechanism, substance accumulation, and the adaption to dehydration stress during seed development of *J. curcas*. A lot of miRNAs were predicted to regulate target genes involved in transcription regulation, lipid metabolism and other important biological processes. These miRNAs might be valuable in improving the yield and quality of *J. curcas* seed oil. Most differentially expressed miRNAs were observed in the late stage of seed development, indicating that miRNAs play important roles in the accumulation of organic matter and dehydration stress during seed maturation. However, further studies are necessary to validate the miRNA targets and the roles of miRNAs in the complex regulation mechanism.

## Materials and Methods

### Seed Collection and RNA Isolation

Fruits from *J. curcas* L. trees grown in Xishuangbanna Tropical Botanical Garden (21°54′ N, 101°46′ E, 580 m a.s.l.,) located in Mengla County, Yunnan Province, China, were collected randomly from 6 plants at 5–10 (young seeds, with seed length less than 10 mm), 12–20 (intermediate seeds, with seed length in the range of 13 to 17 mm) and 25–35 (mature seeds, with seed length more than 20 mm) days after flower opening (DAF) (Supplementary Fig. S1). Three independent biological replicates of developing seeds at each stage were collected and immediately frozen in liquid nitrogen and then stored at −80 °C. Total RNA was isolated from a pool of seeds from each stage with Trizol (Invitrogen, CA, USA), according to the manufacturer’s protocol.

### sRNA Library Construction and Deep Sequencing

Total RNA (1.5 μg per sample) was sent to Biomarker Technologies (Beijing, China) for sRNA library construction and deep sequencing. The sRNA libraries were generated using NEBNext^®^ Ultra^TM^small RNA Sample Library Prep Kit for Illumina^®^ (NEB, USA) following the manufacturer’s recommendations. In brief, RNA bands corresponding to a size range of 16–30 nt were separated and purified from the acrylamide gel. The sRNA molecules ligated with 5′ and 3′ adaptors were used for reverse transcription and subsequent PCR. Small RNA samples were sequenced using Illumina HiSeq. 2500 platform (San Diego, CA, USA) and single-end reads were generated. Three independent sRNA libraries were constructed from each of the three stages of developing seeds.

### Sequence Data Analysis

The reads generated by sRNA-sequencing (named raw reads) were preprocessed with Fastx-toolkit (http://hannonlab.cshl.edu/fastx_toolkit/). First, the raw reads were processed to remove adaptors. Then reads with >20% bases <Q30, or with N base >10%, or with ployA/T/C/G, or read length < 18 nt or >30 nt, were filtered. The remaining reads named clean reads were used for further analysis. sRNAs derived from Viridiplantae rRNAs, scRNA, snRNAs, snoRNAs, tRNA, and repetitive elements (from the SILVA rRNA, Rfam, tRNAdb, and Repbase databases) were identified by mapping with Bowtie v 0.12.9 without mismatches^76^. The remaining reads named unannotated reads without rRNA, scRNA, snRNA, snoRNA, tRNA, or repetitive element were used for miRNA identification.

### miRNA Identification

The unannotated reads containing miRNAs were processed for miRNA identification by miRDeep2 v 2.0.0.5^31^ with modified parameters (sRNAs ≤ 15 hits; 250 nt flanking sequences) for plant miRNA characters^30^. In brief, the analysis procedure of miRDeep2 were: (1) The unannotated reads were aligned to the *Jatropha* genome sequences using Bowtie v 0.12.9 with perfect matches^76^, and only sRNAs with no more than 15 hits were kept and their flanking sequences (250 nt on each side) on the genome were extracted as candidate miRNA precursors, to satisfy the parameters of miRNAs in plant species as described^25^; (2) the precursors were folded in silico using RNAfold (v2.1.7; default parameters) to find the precursors with expected structures; and (3) the unannotated reads were aligned to the precursors, and statistical analyses of read distributions were generated for true miRNA evaluation. To select high confidence miRNAs, more strict criteria were adopted to check the miRDeep2 output figures manually^25,26,32^. Candidate miRNAs were then aligned to the known miRNAs from miRBase 21.0. The miRNAs shared homology with known miRNAs (allowed two mismatches) were identified as conserved miRNA, and those shared no homology to all known sequences in miRBase, novel miRNAs. Isoform miRNAs (isomiRNAs) were analyzed using isomiRID v 0.53 (using default parameters except for cutoff value 100; RangeSize: 18~26)^77^. The reads that were perfectly mapped in the annotated miRNA precursors but not representing the identified miRNA mature and star sequences, shifted not more than four positions from their original mature or star miRNA 5′ position (allowing one mismatch in the middle), and had a total number of reads not less than 50% of the total reads of their reference miRNA or had a total reads of more than 10,000, were considered as isoform miRNAs in *Jatropha*.

### miRNA Target Prediction

The miRNA target prediction was performed by aligning the mature miRNA sequences against *J. curcas* RNA sequences, with Targetfinder v 1.6 using a strict prediction score cutoff value 3 (default = 4)^36^ and psRNAtarget (2017 release) using default parameters^35^. Target annotation was performed by using BLASTX (blast 2.2.25+; -evalue 1e-6, -word_size 6, and -max_target_seqs. 10) based on sequence similarity with genes from the NCBI nr, Swiss-Prot/Uniprot protein databases (http://www.expasy.ch/sprot), and Clusters of Orthologous Groups of proteins (COG) database (http://www.ncbi.nlm.nih.gov/COG/). Blast2GO was employed to obtain Gene Ontology (GO) annotation (http://www.geneontology.org) according to molecular function, biological process, and cellular component ontology with default parameters to evaluate their putative functions^78^. For those miRNA targets could not be mapped by Blast2GO, eggNOG (v4.5) was used to characterize their functional terms^79^.

### In Silico Expression Analysis of miRNAs

The read count for each miRNA was obtained from each sample by mapping sRNA back onto the precursor sequence using the quantifier module of miRDeep2 v 2.0.0.5 with parameters allowing one mismatch and a small window of 2 nt upstream and 5 nt downstream as described by Friedländer *et al*.^31^. To compare the abundances of miRNA between different samples, a normalized read counts named TPM value for each sample was defined as ‘counts of reads mapped to miRNA × 1 000 000’/‘reads mapped to the reference genome’^33,36^. The DESeq2 software provides statistical routines for determining differential expression in digital miRNA expression data using a model based on the negative binomial distribution^80^, and therefore the read counts of miRNAs from different samples were further analyzed using the DESeq2R package (1.14.1) with default parameters. The resulting ‘log_2_ fold change’ represented the base-2 logarithm of fold change of expression levels between any two of young, intermediate, and mature stages; the resulting adjusted *p* values were *p* values adjusted using the Benjamini and Hochberg’s approach for controlling the false discovery rate (FDR). The miRNAs whose expression levels between any two of the three different developmental stages varied significantly (|log_2_ fold change| >1; FDR adjusted *p* < 0.05) were assigned as differentially expressed ones.

### RT-qPCR

RT-qPCR was performed following previously reported procedures^81^. In brief, small RNAs (<200 bp) were extracted with the BioTeKe miRNA extraction kit (BioTeKe Corporation, China). The extracted small RNAs were treated with DNase I and polyadenylated by poly(A) polymerase (New England Biolabs) following by reverse transcription using SuperScript™ III reverse transcriptase (Invitrogen) according to the manufacturer’s instructions. Real-time PCR was performed following a standard SYBR Premix Ex Taq II (TaKaRa) protocol. PCRs were performed in ABI StepOne (USA) as follows: 95 °C for 5 min; then followed by 40 cycles of the normal program (95 °C for 10 s, 60 °C for 30 s). The differences in gene expression were calculated using the 2^−ΔΔCt^ analysis method^82^, and the transcription levels were determined by relative quantification using the 5.8 S RNA as internal reference gene and the young seeds as control. All primers use in RT-qPCR experiments were listed in Supplementary Table S6.

## Supplementary information

Supplementary Information

Table-S1

Table-S2

Table-S3

Table-S4

Table-S5

Table-S6

## Data Availability

All sequencing data were deposited in the NCBI Sequence Read Archive under ID SRA616450. All data generated or analyzed during this study are included in this published article and its Supplementary Information files.
