# Supplementary material for: Identifying High Confidence microRNAs in the Developing Seeds of Jatropha curcas
Source: Sci Rep. 2019 Mar 14;9:4510. doi: 10.1038/s41598-019-41189-y (PMC6418140; doi:10.1038/s41598-019-41189-y)
Supplement: Supplementary file 1 — Supplementary Information [file 41598_2019_41189_MOESM1_ESM.pdf]

Identifying High Confidence microRNAs in the Developing Seeds of *Jatropha curcas*

Mingfeng Yang[1 §], Heshu Lu[1 §], Feiyan Xue[1], Lanqing Ma[1*]

[1] Key Laboratory of Urban Agriculture (North China) Ministry of Agriculture, Beijing University of Agriculture, Beijing 102206, China
[*]lqma@bua.edu.cn
[§] These authors contributed equally to this work

Mingfeng YANG
mfyang@bua.edu.cn

Heshu LU
hslv@bua.edu.cn

Feiyan XUE
fyxue@bua.edu.cn

Lanqing MA
lqma@bua.edu.cn

[*] Corresponding author: Lanqing MA
Key Laboratory of Urban Agriculture (North China) Ministry of Agriculture, Beijing University of Agriculture, Beijing 102206, China
E-mail: lqma@bua.edu.cn
or mfyang@bua.edu.cn

Legends of Supplementary Figures

Supplementary Figure S1. Characterization of *J. curcas* seed development. Developing seeds were divided into three different development stages, i.e., young (groups 1-4), intermediate (groups 6-7) and mature (groups 8-9) seeds (a). Values are the mean ± s.d. of three biological replicates of seed length (b), seed weight (c), and water content (d).

Supplementary Figure S2. The structures of miRNA precursors in *J. curcas*. The stem-loop structures of miRNA precursors are shown on the right of each miRNA Provisional ID. Mature miRNAs and star miRNAs are labeled in red and purple, respectively. These figures were from miRDeep2 software.

Supplementary Figure S3. The biological process, molecular function, and cellular component category of Gene Ontology (GO) annotation for miRNA targets. X-axis indicates the sequence count of miRNA targets (Seqs); y-axis represents GO category.

Supplementary Figure S4. RT-qPCR validatioin for the differentially expressed miRNAs. The expression level of each miRNA at young stage of seed development is set to 1. The miRNAs whose expression levels changed significantly ($p < 0.05$, t-test) compared with young stage were assigned as differentially expressed miRNAs (indicated by *). Values are the mean ± s.d. of three biological replicates. nMIR002 and nMIR013 were not shown as they could not be detected in young and intermediate seeds.

Supplementary Table S1. Size distribution of small RNA in nine sRNA libraries of *J. curcas* developing seeds.

Supplementary Table S2. High confidence miRNAs identified according to the rules of miRdeep2, Meyers *et al*., and Kozomara and Griffiths-Jones.

Supplementary Table S3. Function annotation of miRNA targets.

Supplementary Table S4. Read counts, TPM value, and $\log_2$ (fold change) of 93 high confidence miRNAs in 9 small RNA libraries of *J. curcas* developing seeds.

Supplementary Table S5. Read counts, TPM value and $\log_2$ (fold change) of miRNA isoforms in 9 sRNA libraries of *J. curcas* developing seeds.

Supplementary Table S6. RT-qPCR primers for validation of the differentially expressed miRNAs.

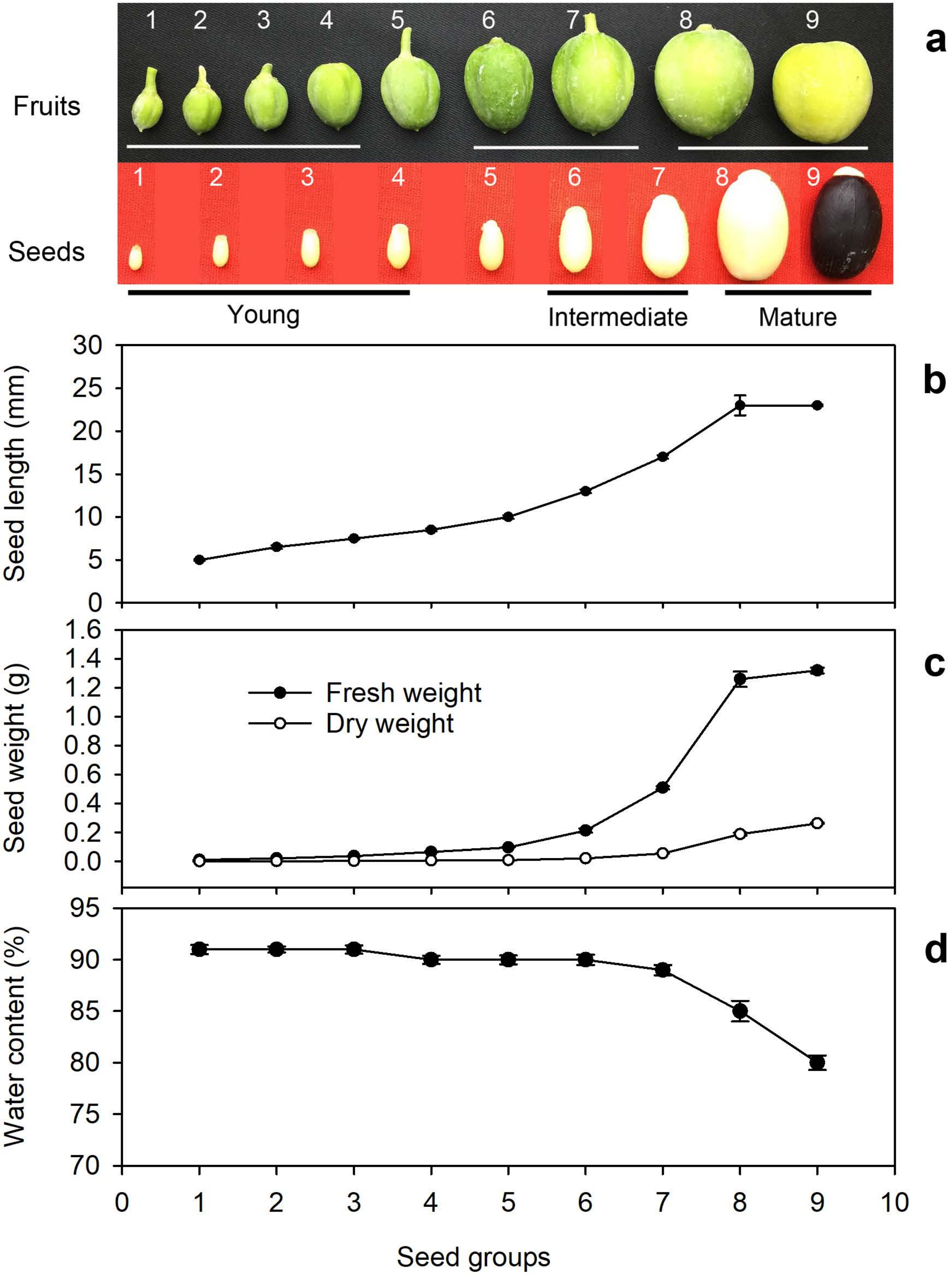
a
Fruits
Seeds
1 2 3 4 5 6 7 8 9
Young
Intermediate
Mature
b
Seed length (mm)
c
Seed weight (g)
Fresh weight
Dry weight
d
Water content (%)
Seed groups

Provisional ID	: conservative_Jcr4S00005_60
Score total	: 849.2
Score for star read(s)	: 3.9
Score for read counts	: 840.3
Score for mfe	: 0.4
Score for randfold	: 1.6
Score for cons. seed	: 3
Total read count	: 1660
Mature read count	: 1659
Loop read count	: 0
Star read count	: 1

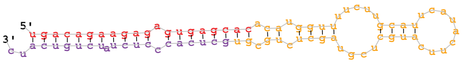

Provisional ID	: conservative_Jcr4S00009_119
Score total	: 1296.2
Score for star read(s)	: 3.9
Score for read counts	: 1287.4
Score for mfe	: 0.2
Score for randfold	: 1.6
Score for cons. seed	: 3
Total read count	: 2537
Mature read count	: 2357
Loop read count	: 4
Star read count	: 176

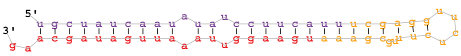

Provisional ID	: conservative_Jcr4S00022_291
Score total	: 4.6
Score for star read(s)	: -1.3
Score for read counts	: 0
Score for mfe	: 1.3
Score for randfold	: 1.6
Score for cons. seed	: 3
Total read count	: 383
Mature read count	: 371
Loop read count	: 0
Star read count	: 12

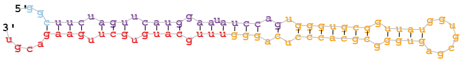

Provisional ID	: conservative_Jcr4S00036_498
Score total	: 534.4
Score for star read(s)	: 3.9
Score for read counts	: 525.8
Score for mfe	: 0.1
Score for randfold	: 1.6
Score for cons. seed	: 3
Total read count	: 1043
Mature read count	: 1041
Loop read count	: 0
Star read count	: 2

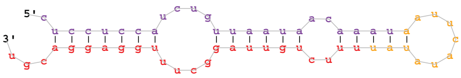

Provisional ID	: conservative_Jcr4S00081_959
Score total	: 2559.5
Score for star read(s)	: 3.9
Score for read counts	: 2548.7
Score for mfe	: 2.3
Score for randfold	: 1.6
Score for cons. seed	: 3
Total read count	: 5011
Mature read count	: 4062
Loop read count	: 89
Star read count	: 860

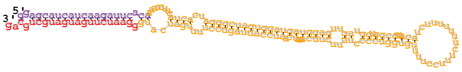

Provisional ID	: conservative_Jcr4S00085_1032
Score total	: 2231.5
Score for star read(s)	: 3.9
Score for read counts	: 2220.4
Score for mfe	: 2.6
Score for randfold	: 1.6
Score for cons. seed	: 3
Total read count	: 4367
Mature read count	: 4348
Loop read count	: 16
Star read count	: 3

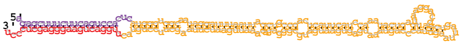

Provisional ID	: conservative_Jcr4S00096_1114
Score total	: 154.3
Score for star read(s)	: 3.9
Score for read counts	: 144.9
Score for mfe	: 0.9
Score for randfold	: 1.6
Score for cons. seed	: 3
Total read count	: 296
Mature read count	: 285
Loop read count	: 4
Star read count	: 7

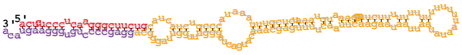

Provisional ID	: conservative_Jcr4S00108_1238
Score total	: 192162.6
Score for star read(s)	: 3.9
Score for read counts	: 192152.7
Score for mfe	: 1.4
Score for randfold	: 1.6
Score for cons. seed	: 3
Total read count	: 376911
Mature read count	: 357950
Loop read count	: 122
Star read count	: 18839

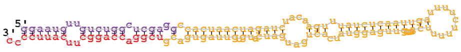

Provisional ID	: conservative_Jcr4S00115_1332
Score total	: 164.3
Score for star read(s)	: 3.9
Score for read counts	: 153.6
Score for mfe	: 2.2
Score for randfold	: 1.6
Score for cons. seed	: 3
Total read count	: 313
Mature read count	: 303
Loop read count	: 0
Star read count	: 10

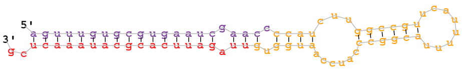

Provisional ID	: conservative_Jcr4S00122_1408
Score total	: 54026.8
Score for star read(s)	: 3.9
Score for read counts	: 54015.6
Score for mfe	: 2.7
Score for randfold	: 1.6
Score for cons. seed	: 3
Total read count	: 105961
Mature read count	: 105324
Loop read count	: 1
Star read count	: 636

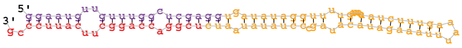

Provisional ID	: conservative_Jcr4S00138_1561
Score total	: 3.4
Score for star read(s)	: -1.3
Score for read counts	: 0
Score for mfe	: 0.1
Score for randfold	: 1.6
Score for cons. seed	: 3
Total read count	: 343541
Mature read count	: 343541
Loop read count	: 0
Star read count	: 0

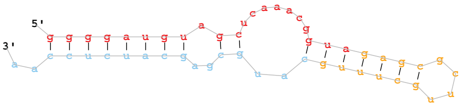

Provisional ID	: conservative_Jcr4S00202_2119
Score total	: 2856
Score for star read(s)	: 3.9
Score for read counts	: 2846.5
Score for mfe	: 1
Score for randfold	: 1.6
Score for cons. seed	: 3
Total read count	: 5595
Mature read count	: 5550
Loop read count	: 0
Star read count	: 45

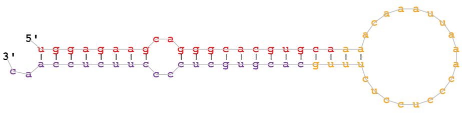

Provisional ID	: conservative_Jcr4S00300_2981
Score total	: 2611.4
Score for star read(s)	: 3.9
Score for read counts	: 2599.2
Score for mfe	: 3.7
Score for randfold	: 1.6
Score for cons. seed	: 3
Total read count	: 5110
Mature read count	: 3609
Loop read count	: 54
Star read count	: 1447

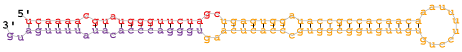

Provisional ID	: conservative_Jcr4S00313_3087
Score total	: 12262.7
Score for star read(s)	: 3.9
Score for read counts	: 12253.3
Score for mfe	: 0.9
Score for randfold	: 1.6
Score for cons. seed	: 3
Total read count	: 24046
Mature read count	: 12785
Loop read count	: 13
Star read count	: 11248

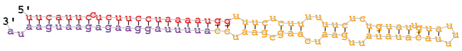

Provisional ID	: conservative_Jcr4S00358_3373
Score total	: 182683.7
Score for star read(s)	: 3.9
Score for read counts	: 182674.5
Score for mfe	: 0.7
Score for randfold	: 1.6
Score for cons. seed	: 3
Total read count	: 358320
Mature read count	: 358187
Loop read count	: 10
Star read count	: 123

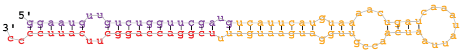

Provisional ID	: conservative_Jcr4S00387_3594
Score total	: 364.7
Score for star read(s)	: 3.9
Score for read counts	: 355
Score for mfe	: 1.2
Score for randfold	: 1.6
Score for cons. seed	: 3
Total read count	: 708
Mature read count	: 562
Loop read count	: 2
Star read count	: 144

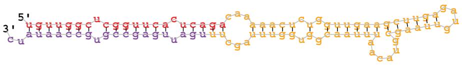

Provisional ID	: conservative_Jcr4S00585_4867
Score total	: 190.1
Score for star read(s)	: 3.9
Score for read counts	: 180.6
Score for mfe	: 1
Score for randfold	: 1.6
Score for cons. seed	: 3
Total read count	: 366
Mature read count	: 311
Loop read count	: 2
Star read count	: 53

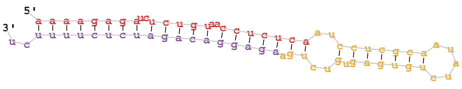

Provisional ID	: conservative_Jcr4S00711_5751
Score total	: 1181.6
Score for star read(s)	: 3.9
Score for read counts	: 1171.7
Score for mfe	: 1.4
Score for randfold	: 1.6
Score for cons. seed	: 3
Total read count	: 2310
Mature read count	: 2095
Loop read count	: 2
Star read count	: 213

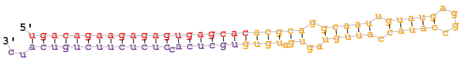

Provisional ID	: conservative_Jcr4S00929_7068
Score total	: 3992.8
Score for star read(s)	: 3.9
Score for read counts	: 3983.4
Score for mfe	: 0.9
Score for randfold	: 1.6
Score for cons. seed	: 3
Total read count	: 7825
Mature read count	: 6432
Loop read count	: 4
Star read count	: 1389

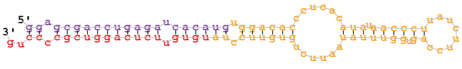

Provisional ID	: conservative_Jcr4S00953_7199
Score total	: 1240.5
Score for star read(s)	: 3.9
Score for read counts	: 1228.3
Score for mfe	: 3.7
Score for randfold	: 1.6
Score for cons. seed	: 3
Total read count	: 2421
Mature read count	: 2371
Loop read count	: 3
Star read count	: 47

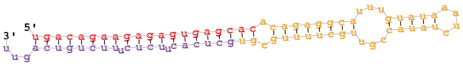

Provisional ID	: conservative_Jcr4S00953_7200
Score total	: 3.4
Score for star read(s)	: -1.3
Score for read counts	: 0
Score for mfe	: 0.1
Score for randfold	: 1.6
Score for cons. seed	: 3
Total read count	: 2374
Mature read count	: 2371
Loop read count	: 0
Star read count	: 3

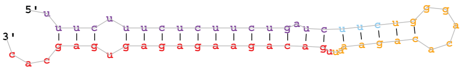

Provisional ID	: conservative_Jcr4S01025_7637
Score total	: 391.1
Score for star read(s)	: 3.9
Score for read counts	: 382.5
Score for mfe	: 0.1
Score for randfold	: 1.6
Score for cons. seed	: 3
Total read count	: 762
Mature read count	: 752
Loop read count	: 6
Star read count	: 4

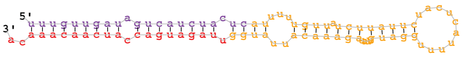

Provisional ID	: conservative_Jcr4S01030_7684
Score total	: 3.4
Score for star read(s)	: -1.3
Score for read counts	: 0
Score for mfe	: 0.1
Score for randfold	: 1.6
Score for cons. seed	: 3
Total read count	: 1678
Mature read count	: 1677
Loop read count	: 0
Star read count	: 1

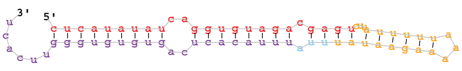

Provisional ID	: conservative_Jcr4S01076_7953
Score total	: 192477.8
Score for star read(s)	: 3.9
Score for read counts	: 192468.3
Score for mfe	: 1
Score for randfold	: 1.6
Score for cons. seed	: 3
Total read count	: 377530
Mature read count	: 377349
Loop read count	: 1
Star read count	: 180

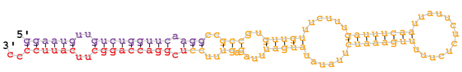

Provisional ID : conservative_Jcr4S01134_8374
Score total : 2865
Score for star read(s) : 3.9
Score for read counts : 2854.6
Score for mfe : 1.9
Score for randfold : 1.6
Score for cons. seed : 3
Total read count : 5611
Mature read count : 5565
Loop read count : 1
Star read count : 45

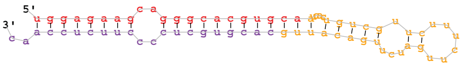

Provisional ID : conservative_Jcr4S01201_8767
Score total : 386151.3
Score for star read(s) : 3.9
Score for read counts : 386139.6
Score for mfe : 3.1
Score for randfold : 1.6
Score for cons. seed : 3
Total read count : 757408
Mature read count : 756527
Loop read count : 334
Star read count : 547

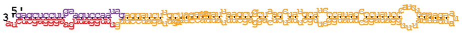

Provisional ID : conservative_Jcr4S01437_9860
Score total : 7.2
Score for star read(s) : -1.3
Score for read counts : 0
Score for mfe : 3.9
Score for randfold : 1.6
Score for cons. seed : 3
Total read count : 2909
Mature read count : 2847
Loop read count : 62
Star read count : 0

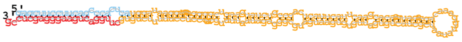

Provisional ID : conservative_Jcr4S01644_10817
Score total : 150951.1
Score for star read(s) : 3.9
Score for read counts : 150942.5
Score for mfe : 0.1
Score for randfold : 1.6
Score for cons. seed : 3
Total read count : 296079
Mature read count : 290091
Loop read count : 0
Star read count : 5988

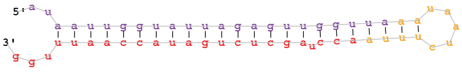

Provisional ID : conservative_Jcr4S01890_12031
Score total : 3.6
Score for star read(s) : -1.3
Score for read counts : 0
Score for mfe : 0.3
Score for randfold : 1.6
Score for cons. seed : 3
Total read count : 9572
Mature read count : 9570
Loop read count : 2
Star read count : 0

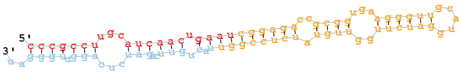

Provisional ID : conservative_Jcr4S01894_12048
Score total : 6077.1
Score for star read(s) : 3.9
Score for read counts : 6067
Score for mfe : 1.5
Score for randfold : 1.6
Score for cons. seed : 3
Total read count : 11912
Mature read count : 11182
Loop read count : 18
Star read count : 712

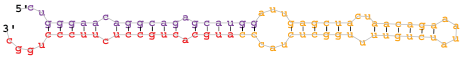

Provisional ID : conservative_Jcr4S01927_12218
Score total : 13577.3
Score for star read(s) : 3.9
Score for read counts : 13564.5
Score for mfe : 4.2
Score for randfold : 1.6
Score for cons. seed : 3
Total read count : 26618
Mature read count : 22677
Loop read count : 1
Star read count : 3940

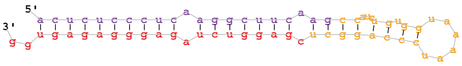

Provisional ID : conservative_Jcr4S02027_12753
Score total : 336.6
Score for star read(s) : 3.9
Score for read counts : 327.9
Score for mfe : 0.2
Score for randfold : 1.6
Score for cons. seed : 3
Total read count : 655
Mature read count : 507
Loop read count : 4
Star read count : 144

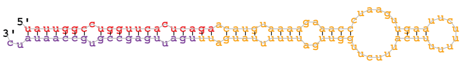

Provisional ID : conservative_Jcr4S02209_13549
Score total : 405.2
Score for star read(s) : 3.9
Score for read counts : 394.2
Score for mfe : 2.4
Score for randfold : 1.6
Score for cons. seed : 3
Total read count : 785
Mature read count : 617
Loop read count : 1
Star read count : 167

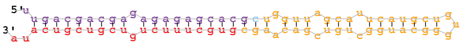

Provisional ID : conservative_Jcr4S02434_14511
Score total : 865.7
Score for star read(s) : 3.9
Score for read counts : 860.7
Score for mfe : 0.3
Score for randfold : -2.2
Score for cons. seed : 3
Total read count : 1700
Mature read count : 1593
Loop read count : 49
Star read count : 58

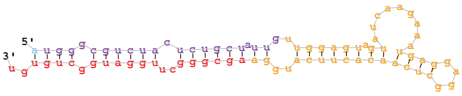

Provisional ID : conservative_Jcr4S02957_16589
Score total : 322.6
Score for star read(s) : 3.9
Score for read counts : 313.7
Score for mfe : 0.5
Score for randfold : 1.6
Score for cons. seed : 3
Total read count : 627
Mature read count : 617
Loop read count : 7
Star read count : 3

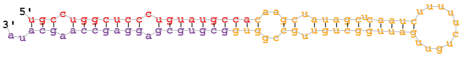

Provisional ID : conservative_Jcr4S02969_16626
Score total : 1203
Score for star read(s) : 3.9
Score for read counts : 1198.2
Score for mfe : 0
Score for randfold : -2.2
Score for cons. seed : 3
Total read count : 2362
Mature read count : 2342
Loop read count : 0
Star read count : 20

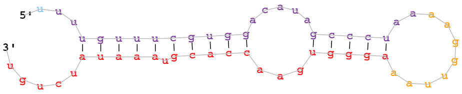

Provisional ID : conservative_Jcr4S03072_16999
Score total : 978.5
Score for star read(s) : 3.9
Score for read counts : 968.8
Score for mfe : 1.2
Score for randfold : 1.6
Score for cons. seed : 3
Total read count : 1912
Mature read count : 1905
Loop read count : 2
Star read count : 5

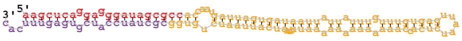

Provisional ID : conservative_Jcr4S03224_17490
Score total : 4
Score for star read(s) : -1.3
Score for read counts : 0
Score for mfe : 0.7
Score for randfold : 1.6
Score for cons. seed : 3
Total read count : 1356
Mature read count : 1356
Loop read count : 0
Star read count : 0

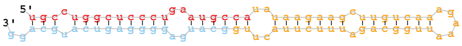

Provisional ID : conservative_Jcr4S03229_17511
Score total : 1545.9
Score for star read(s) : 3.9
Score for read counts : 1536.7
Score for mfe : 0.6
Score for randfold : 1.6
Score for cons. seed : 3
Total read count : 3026
Mature read count : 2460
Loop read count : 530
Star read count : 36

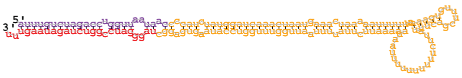

Provisional ID : conservative_Jcr4S03474_18392
Score total : 5092.4
Score for star read(s) : 3.9
Score for read counts : 5083.6
Score for mfe : 0.3
Score for randfold : 1.6
Score for cons. seed : 3
Total read count : 9983
Mature read count : 9827
Loop read count : 1
Star read count : 155

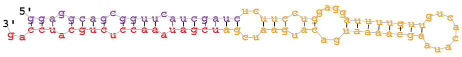

Provisional ID : conservative_Jcr4S03476_18399
Score total : 7.8
Score for star read(s) : -1.3
Score for read counts : 0
Score for mfe : 4.5
Score for randfold : 1.6
Score for cons. seed : 3
Total read count : 1219
Mature read count : 1219
Loop read count : 0
Star read count : 0

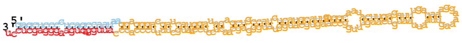

Provisional ID : conservative_Jcr4S03532_18591
Score total : 393.9
Score for star read(s) : 3.9
Score for read counts : 384
Score for mfe : 1.4
Score for randfold : 1.6
Score for cons. seed : 3
Total read count : 765
Mature read count : 642
Loop read count : 1
Star read count : 122

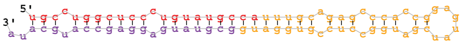

Provisional ID : conservative_Jcr4S03975_19986
Score total : 66541.1
Score for star read(s) : 3.9
Score for read counts : 66531.8
Score for mfe : 0.8
Score for randfold : 1.6
Score for cons. seed : 3
Total read count : 130511
Mature read count : 110328
Loop read count : 24
Star read count : 20159

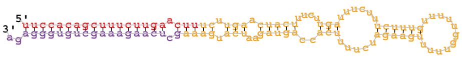

Provisional ID : conservative_Jcr4S03975_19989
Score total : 14968.7
Score for star read(s) : 3.9
Score for read counts : 14958.9
Score for mfe : 1.2
Score for randfold : 1.6
Score for cons. seed : 3
Total read count : 29353
Mature read count : 23460
Loop read count : 0
Star read count : 5893

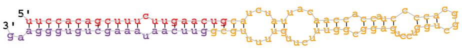

Provisional ID : conservative_Jcr4S04133_20466
Score total : 3.3
Score for star read(s) : -1.3
Score for read counts : 0
Score for mfe : 0
Score for randfold : 1.6
Score for cons. seed : 3
Total read count : 516
Mature read count : 416
Loop read count : 100
Star read count : 0

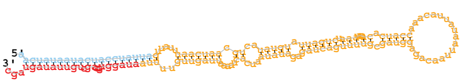

Provisional ID : conservative_Jcr4S04196_20632
Score total : 537
Score for star read(s) : 3.9
Score for read counts : 527.8
Score for mfe : 0.7
Score for randfold : 1.6
Score for cons. seed : 3
Total read count : 1047
Mature read count : 720
Loop read count : 1
Star read count : 326

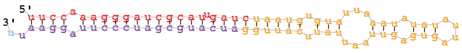

Provisional ID : conservative_Jcr4S04205_20642
Score total : 2234.3
Score for star read(s) : 3.9
Score for read counts : 2221.9
Score for mfe : 3.8
Score for randfold : 1.6
Score for cons. seed : 3
Total read count : 4370
Mature read count : 4304
Loop read count : 63
Star read count : 3

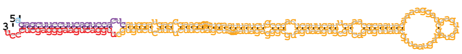

Provisional ID : conservative_Jcr4S04290_20890
Score total : 1348.8
Score for star read(s) : 3.9
Score for read counts : 1344
Score for mfe : 0
Score for randfold : -2.2
Score for cons. seed : 3
Total read count : 2648
Mature read count : 2208
Loop read count : 427
Star read count : 13

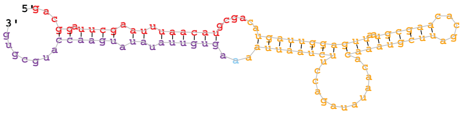

Provisional ID	: conservative_Jcr4S04439_21322
Score total	: 186964.3
Score for star read(s)	: 3.9
Score for read counts	: 186955
Score for mfe	: 0.8
Score for randfold	: 1.6
Score for cons. seed	: 3
Total read count	: 366716
Mature read count	: 366674
Loop read count	: 1
Star read count	: 41

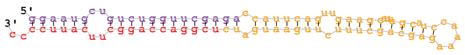

Provisional ID	: conservative_Jcr4S05072_23150
Score total	: 393.6
Score for star read(s)	: 3.9
Score for read counts	: 385
Score for mfe	: 0.1
Score for randfold	: 1.6
Score for cons. seed	: 3
Total read count	: 767
Mature read count	: 766
Loop read count	: 0
Star read count	: 1

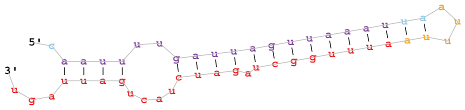

Provisional ID	: conservative_Jcr4S05692_24674
Score total	: 7.5
Score for star read(s)	: -1.3
Score for read counts	: 0
Score for mfe	: 4.2
Score for randfold	: 1.6
Score for cons. seed	: 3
Total read count	: 3774
Mature read count	: 3770
Loop read count	: 4
Star read count	: 0

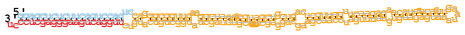

Provisional ID	: conservative_Jcr4S06267_26017
Score total	: 10072.3
Score for star read(s)	: 3.9
Score for read counts	: 10063.6
Score for mfe	: 0.2
Score for randfold	: 1.6
Score for cons. seed	: 3
Total read count	: 19751
Mature read count	: 19736
Loop read count	: 0
Star read count	: 15

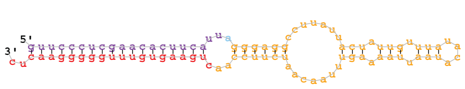

Provisional ID	: conservative_Jcr4S06500_26595
Score total	: 717.3
Score for star read(s)	: 3.9
Score for read counts	: 707.8
Score for mfe	: 1.1
Score for randfold	: 1.6
Score for cons. seed	: 3
Total read count	: 1400
Mature read count	: 1399
Loop read count	: 0
Star read count	: 1

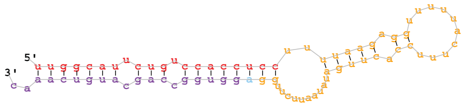

Provisional ID	: conservative_Jcr4S06778_27228
Score total	: 718.2
Score for star read(s)	: 3.9
Score for read counts	: 713.4
Score for mfe	: 0.1
Score for randfold	: -2.2
Score for cons. seed	: 3
Total read count	: 1411
Mature read count	: 1408
Loop read count	: 2
Star read count	: 1

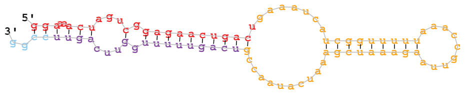

Provisional ID	: conservative_Jcr4S07008_27722
Score total	: 718.2
Score for star read(s)	: 3.9
Score for read counts	: 713.4
Score for mfe	: 0.1
Score for randfold	: -2.2
Score for cons. seed	: 3
Total read count	: 1411
Mature read count	: 1408
Loop read count	: 2
Star read count	: 1

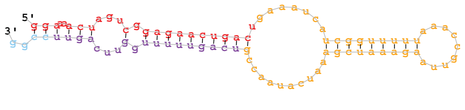

Provisional ID	: conservative_Jcr4S08181_29922
Score total	: 153.6
Score for star read(s)	: 3.9
Score for read counts	: 144.9
Score for mfe	: 0.2
Score for randfold	: 1.6
Score for cons. seed	: 3
Total read count	: 296
Mature read count	: 295
Loop read count	: 0
Star read count	: 1

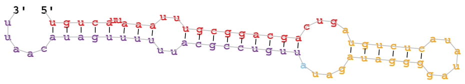

Provisional ID	: conservative_Jcr4S09210_31379
Score total	: 3.5
Score for star read(s)	: -1.3
Score for read counts	: 0
Score for mfe	: 0.2
Score for randfold	: 1.6
Score for cons. seed	: 3
Total read count	: 1971
Mature read count	: 1948
Loop read count	: 16
Star read count	: 7

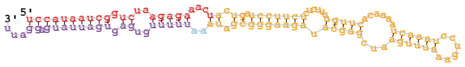

Provisional ID	: conservative_Jcr4S09730_32175
Score total	: 49059.8
Score for star read(s)	: 3.9
Score for read counts	: 49050.4
Score for mfe	: 0.9
Score for randfold	: 1.6
Score for cons. seed	: 3
Total read count	: 96222
Mature read count	: 95334
Loop read count	: 2
Star read count	: 886

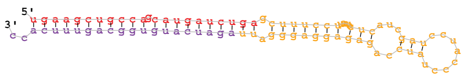

Provisional ID	: conservative_Jcr4S09898_32449
Score total	: 11779.7
Score for star read(s)	: 3.9
Score for read counts	: 11771
Score for mfe	: 0.2
Score for randfold	: 1.6
Score for cons. seed	: 3
Total read count	: 23100
Mature read count	: 23073
Loop read count	: 5
Star read count	: 22

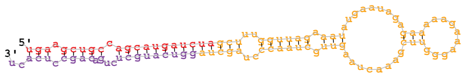

Provisional ID	: conservative_Jcr4S12814_35898
Score total	: 3.4
Score for star read(s)	: -1.3
Score for read counts	: 0
Score for mfe	: 0.1
Score for randfold	: 1.6
Score for cons. seed	: 3
Total read count	: 343541
Mature read count	: 343541
Loop read count	: 0
Star read count	: 0

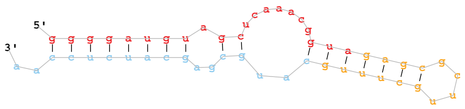

Provisional ID	: conservative_Jcr4S04450_21362
Score total	: 555.8
Score for star read(s)	: 3.9
Score for read counts	: 546.7
Score for mfe	: 0.6
Score for randfold	: 1.6
Score for cons. seed	: 3
Total read count	: 1084
Mature read count	: 843
Loop read count	: 2
Star read count	: 239

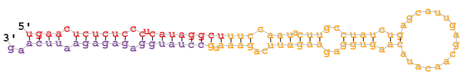

Provisional ID	: conservative_Jcr4S05481_24205
Score total	: 865.9
Score for star read(s)	: 3.9
Score for read counts	: 855.6
Score for mfe	: 1.8
Score for randfold	: 1.6
Score for cons. seed	: 3
Total read count	: 1690
Mature read count	: 1659
Loop read count	: 0
Star read count	: 31

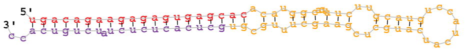

Provisional ID	: conservative_Jcr4S06221_25930
Score total	: 5975
Score for star read(s)	: 3.9
Score for read counts	: 5966.1
Score for mfe	: 0.4
Score for randfold	: 1.6
Score for cons. seed	: 3
Total read count	: 11714
Mature read count	: 11671
Loop read count	: 6
Star read count	: 37

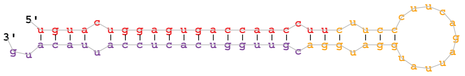

Provisional ID	: conservative_Jcr4S06267_26022
Score total	: 10075.7
Score for star read(s)	: 3.9
Score for read counts	: 10066.6
Score for mfe	: 0.6
Score for randfold	: 1.6
Score for cons. seed	: 3
Total read count	: 19757
Mature read count	: 19752
Loop read count	: 0
Star read count	: 5

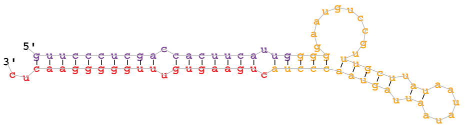

Provisional ID	: conservative_Jcr4S06635_26892
Score total	: 4618.4
Score for star read(s)	: 3.9
Score for read counts	: 4608.9
Score for mfe	: 1
Score for randfold	: 1.6
Score for cons. seed	: 3
Total read count	: 9052
Mature read count	: 4854
Loop read count	: 12
Star read count	: 4186

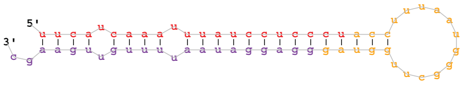

Provisional ID	: conservative_Jcr4S06928_27533
Score total	: 1044.4
Score for star read(s)	: 3.9
Score for read counts	: 1032.5
Score for mfe	: 3.4
Score for randfold	: 1.6
Score for cons. seed	: 3
Total read count	: 2037
Mature read count	: 2034
Loop read count	: 0
Star read count	: 3

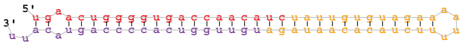

Provisional ID	: conservative_Jcr4S08147_29855
Score total	: 304.6
Score for star read(s)	: 3.9
Score for read counts	: 299.9
Score for mfe	: 0
Score for randfold	: -2.2
Score for cons. seed	: 3
Total read count	: 600
Mature read count	: 480
Loop read count	: 107
Star read count	: 13

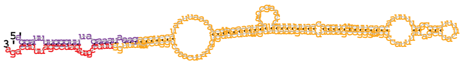

Provisional ID	: conservative_Jcr4S08406_30209
Score total	: 285.2
Score for star read(s)	: 3.9
Score for read counts	: 273.4
Score for mfe	: 3.4
Score for randfold	: 1.6
Score for cons. seed	: 3
Total read count	: 548
Mature read count	: 501
Loop read count	: 2
Star read count	: 45

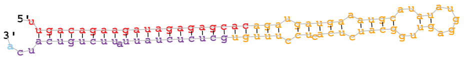

Provisional ID	: conservative_Jcr4S09448_31775
Score total	: 3.4
Score for star read(s)	: -1.3
Score for read counts	: 0
Score for mfe	: 0.1
Score for randfold	: 1.6
Score for cons. seed	: 3
Total read count	: 34262
Mature read count	: 34261
Loop read count	: 1
Star read count	: 0

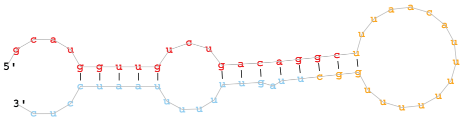

Provisional ID	: conservative_Jcr4S09877_32399
Score total	: 32086.2
Score for star read(s)	: 3.9
Score for read counts	: 32073.2
Score for mfe	: 4.5
Score for randfold	: 1.6
Score for cons. seed	: 3
Total read count	: 62922
Mature read count	: 56751
Loop read count	: 3194
Star read count	: 2977

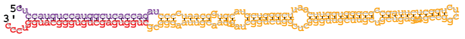

Provisional ID	: conservative_Jcr4S10655_33343
Score total	: 3.3
Score for star read(s)	: -1.3
Score for read counts	: 0
Score for mfe	: 0
Score for randfold	: 1.6
Score for cons. seed	: 3
Total read count	: 358
Mature read count	: 353
Loop read count	: 5
Star read count	: 0

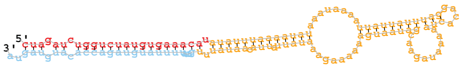

Provisional ID	: conservative_Jcr4S13375_36625
Score total	: 31761.2
Score for star read(s)	: 3.9
Score for read counts	: 31748
Score for mfe	: 4.7
Score for randfold	: 1.6
Score for cons. seed	: 3
Total read count	: 62284
Mature read count	: 56751
Loop read count	: 2556
Star read count	: 2977

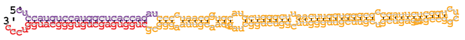

Provisional ID : conservative_Jcr4S13637_36911
Score total : 535.6
Score for star read(s) : 3.9
Score for read counts : 530.9
Score for mfe : 0.1
Score for randfold : -2.2
Score for cons. seed : 3
Total read count : 1053
Mature read count : 1051
Loop read count : 1
Star read count : 1

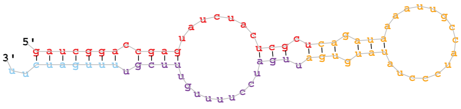

Provisional ID : conservative_Jcr4S15535_38615
Score total : 5969.1
Score for star read(s) : 3.9
Score for read counts : 5960
Score for mfe : 0.6
Score for randfold : 1.6
Score for cons. seed : 3
Total read count : 11702
Mature read count : 11641
Loop read count : 9
Star read count : 52

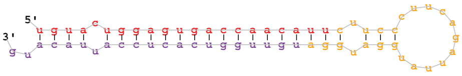

Provisional ID : conservative_Jcr4S17186_40158
Score total : 12153.1
Score for star read(s) : 3.9
Score for read counts : 12144.2
Score for mfe : 0.4
Score for randfold : 1.6
Score for cons. seed : 3
Total read count : 23832
Mature read count : 23708
Loop read count : 0
Star read count : 124

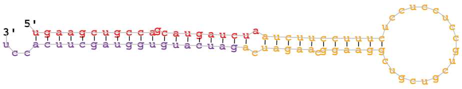

Provisional ID : conservative_Jcr4S21142_42657
Score total : 32086.3
Score for star read(s) : 3.9
Score for read counts : 32073.2
Score for mfe : 4.6
Score for randfold : 1.6
Score for cons. seed : 3
Total read count : 62922
Mature read count : 56751
Loop read count : 3194
Star read count : 2977

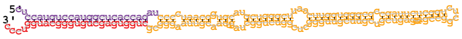

Provisional ID : conservative_Jcr4S21962_43107
Score total : 5969.2
Score for star read(s) : 3.9
Score for read counts : 5960
Score for mfe : 0.7
Score for randfold : 1.6
Score for cons. seed : 3
Total read count : 11702
Mature read count : 11641
Loop read count : 9
Star read count : 52

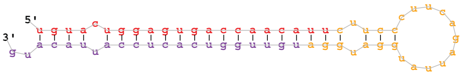

Provisional ID : conservative_Jcr4S26535_45222
Score total : 3.4
Score for star read(s) : -1.3
Score for read counts : 0
Score for mfe : 0.1
Score for randfold : 1.6
Score for cons. seed : 3
Total read count : 616
Mature read count : 616
Loop read count : 0
Star read count : 0

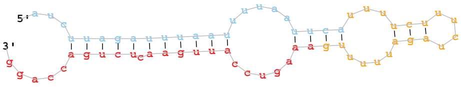

Provisional ID : conservative_Jcr4S27644_45982
Score total : 31139.2
Score for star read(s) : 3.9
Score for read counts : 31126
Score for mfe : 4.8
Score for randfold : 1.6
Score for cons. seed : 3
Total read count : 61064
Mature read count : 56751
Loop read count : 1336
Star read count : 2977

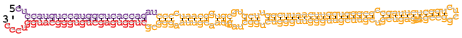

Provisional ID : conservative_Jcr4U31285_47895
Score total : 54027.5
Score for star read(s) : 3.9
Score for read counts : 54015.6
Score for mfe : 3.4
Score for randfold : 1.6
Score for cons. seed : 3
Total read count : 105961
Mature read count : 105324
Loop read count : 1
Star read count : 636

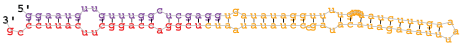

Provisional ID : unconservative_Jcr4S00105_1216
Score total : 905.5
Score for star read(s) : 3.9
Score for read counts : 899.5
Score for mfe : 1.1
Score for randfold : 1.6
Score for cons. seed : -0.6
Total read count : 1776
Mature read count : 1527
Loop read count : 5
Star read count : 244

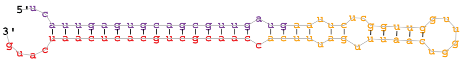

Provisional ID : unconservative_Jcr4S05946_25260
Score total : 489.2
Score for star read(s) : 3.9
Score for read counts : 488
Score for mfe : 0
Score for randfold : -2.2
Score for cons. seed : -0.6
Total read count : 969
Mature read count : 966
Loop read count : 0
Star read count : 3

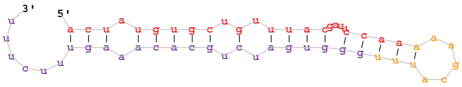

Provisional ID : unconservative_Jcr4S10606_33275
Score total : 300
Score for star read(s) : 3.9
Score for read counts : 298.9
Score for mfe : 0
Score for randfold : -2.2
Score for cons. seed : -0.6
Total read count : 598
Mature read count : 586
Loop read count : 10
Star read count : 2

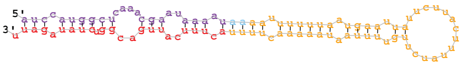

Provisional ID : conservative_Jcr4S14290_37670
Score total : 3.4
Score for star read(s) : -1.3
Score for read counts : 0
Score for mfe : 0.1
Score for randfold : 1.6
Score for cons. seed : 3
Total read count : 616
Mature read count : 616
Loop read count : 0
Star read count : 0

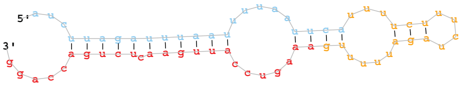

Provisional ID : conservative_Jcr4S16131_39216
Score total : 717.4
Score for star read(s) : 3.9
Score for read counts : 707.8
Score for mfe : 1.2
Score for randfold : 1.6
Score for cons. seed : 3
Total read count : 1400
Mature read count : 1399
Loop read count : 0
Star read count : 1

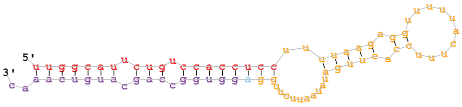

Provisional ID : conservative_Jcr4S17700_40443
Score total : 6077.1
Score for star read(s) : 3.9
Score for read counts : 6067
Score for mfe : 1.6
Score for randfold : 1.6
Score for cons. seed : 3
Total read count : 11912
Mature read count : 11182
Loop read count : 18
Star read count : 712

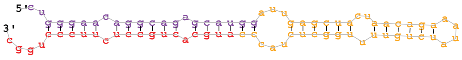

Provisional ID : conservative_Jcr4S21142_42658
Score total : 8.1
Score for star read(s) : -1.3
Score for read counts : 0
Score for mfe : 4.8
Score for randfold : 1.6
Score for cons. seed : 3
Total read count : 62215
Mature read count : 56751
Loop read count : 5382
Star read count : 82

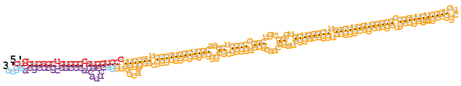

Provisional ID : conservative_Jcr4S22615_43409
Score total : 5975
Score for star read(s) : 3.9
Score for read counts : 5966.1
Score for mfe : 0.4
Score for randfold : 1.6
Score for cons. seed : 3
Total read count : 11714
Mature read count : 11671
Loop read count : 6
Star read count : 37

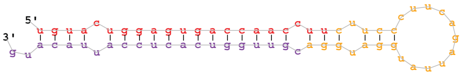

Provisional ID : conservative_Jcr4S27644_45981
Score total : 8.1
Score for star read(s) : -1.3
Score for read counts : 0
Score for mfe : 4.8
Score for randfold : 1.6
Score for cons. seed : 3
Total read count : 62532
Mature read count : 56751
Loop read count : 5665
Star read count : 116

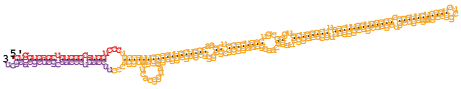

Provisional ID : conservative_Jcr4S28665_46581
Score total : 31761.2
Score for star read(s) : 3.9
Score for read counts : 31748
Score for mfe : 4.7
Score for randfold : 1.6
Score for cons. seed : 3
Total read count : 62284
Mature read count : 56751
Loop read count : 2556
Star read count : 2977

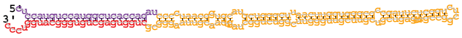

Provisional ID : conservative_Jcr4U31709_48101
Score total : 555.6
Score for star read(s) : 3.9
Score for read counts : 546.7
Score for mfe : 0.4
Score for randfold : 1.6
Score for cons. seed : 3
Total read count : 1084
Mature read count : 843
Loop read count : 2
Star read count : 239

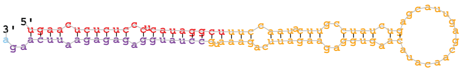

Provisional ID : unconservative_Jcr4S00244_2520
Score total : 166.4
Score for star read(s) : 3.9
Score for read counts : 165.3
Score for mfe : 0
Score for randfold : -2.2
Score for cons. seed : -0.6
Total read count : 336
Mature read count : 332
Loop read count : 0
Star read count : 4

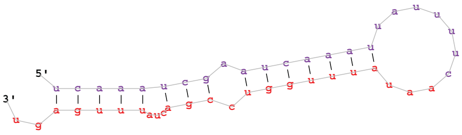

Provisional ID : unconservative_Jcr4S09458_31792
Score total : 1730.9
Score for star read(s) : 3.9
Score for read counts : 1725.9
Score for mfe : 0.1
Score for randfold : 1.6
Score for cons. seed : -0.6
Total read count : 3397
Mature read count : 3394
Loop read count : 2
Star read count : 1

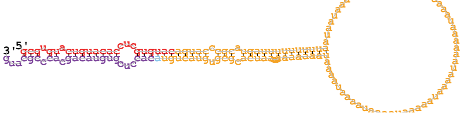

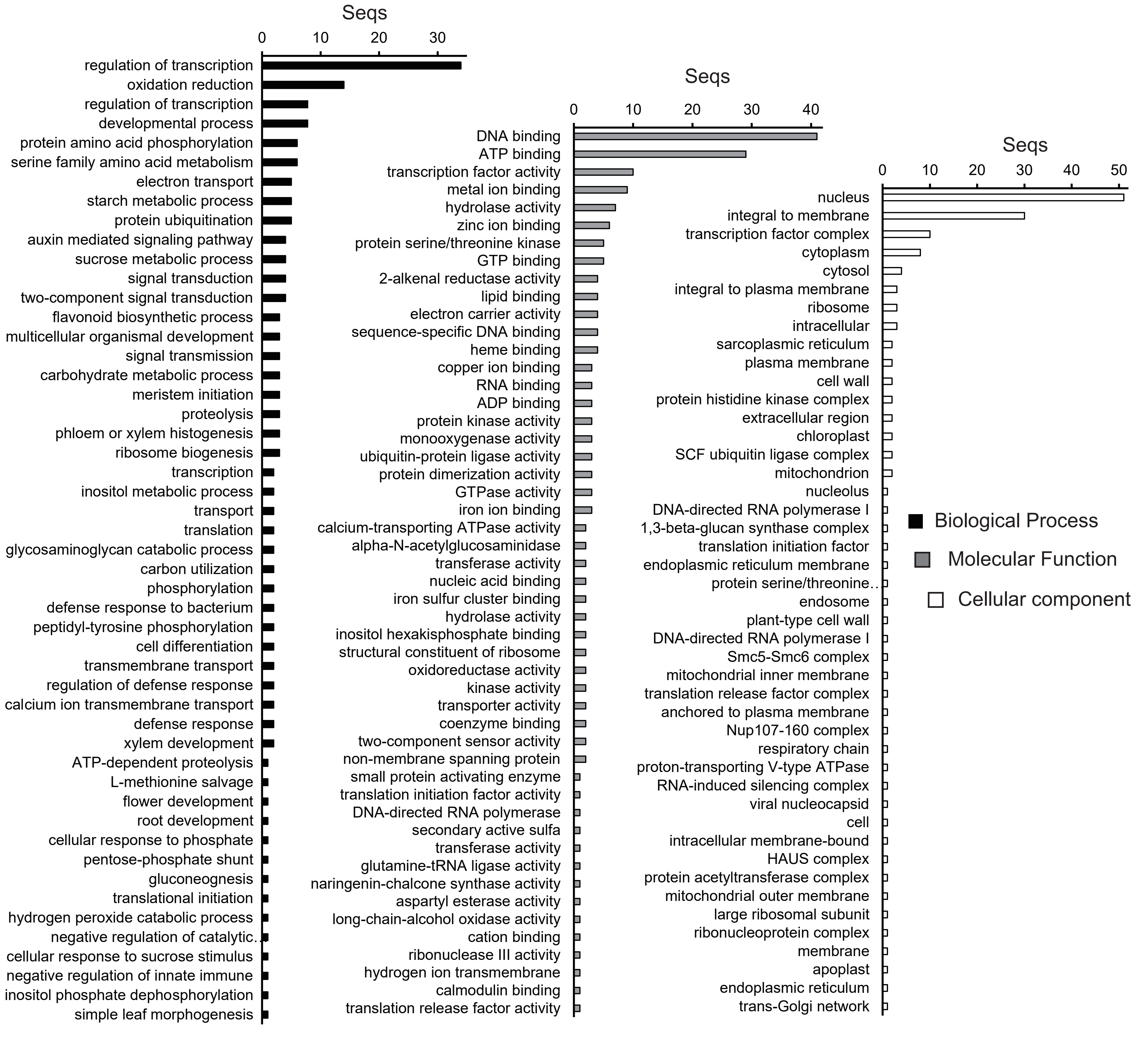
Seqs
0
10
20
30
regulation of transcription
oxidation reduction
regulation of transcription
developmental process
protein amino acid phosphorylation
serine family amino acid metabolism
electron transport
starch metabolic process
protein ubiquitination
auxin mediated signaling pathway
sucrose metabolic process
signal transduction
two-component signal transduction
flavonoid biosynthetic process
multicellular organismal development
signal transmission
carbohydrate metabolic process
meristem initiation
proteolysis
phloem or xylem histogenesis
ribosome biogenesis
transcription
inositol metabolic process
transport
translation
glycosaminoglycan catabolic process
carbon utilization
phosphorylation
defense response to bacterium
peptidyl-tyrosine phosphorylation
cell differentiation
transmembrane transport
regulation of defense response
calcium ion transmembrane transport
defense response
xylem development
ATP-dependent proteolysis
L-methionine salvage
flower development
root development
cellular response to phosphate
pentose-phosphate shunt
gluconeognesis
translational initiation
hydrogen peroxide catabolic process
negative regulation of catalytic…
cellular response to sucrose stimulus
negative regulation of innate immune
inositol phosphate dephosphorylation
simple leaf morphogenesis
Seqs
0
10
20
30
40
DNA binding
ATP binding
transcription factor activity
metal ion binding
hydrolase activity
zinc ion binding
protein serine/threonine kinase
GTP binding
2-alkenal reductase activity
lipid binding
electron carrier activity
sequence-specific DNA binding
heme binding
copper ion binding
RNA binding
ADP binding
protein kinase activity
monooxygenase activity
ubiquitin-protein ligase activity
protein dimerization activity
GTPase activity
iron ion binding
calcium-transporting ATPase activity
alpha-N-acetylglucosaminidase
transferase activity
nucleic acid binding
iron sulfur cluster binding
hydrolase activity
inositol hexakisphosphate binding
structural constituent of ribosome
oxidoreductase activity
kinase activity
transporter activity
coenzyme binding
two-component sensor activity
non-membrane spanning protein
small protein activating enzyme
translation initiation factor activity
DNA-directed RNA polymerase
secondary active sulfa
transferase activity
glutamine-tRNA ligase activity
naringenin-chalcone synthase activity
aspartyl esterase activity
long-chain-alcohol oxidase activity
cation binding
ribonuclease III activity
hydrogen ion transmembrane
calmodulin binding
translation release factor activity
Seqs
0
10
20
30
40
50
nucleus
integral to membrane
transcription factor complex
cytoplasm
cytosol
integral to plasma membrane
ribosome
intracellular
sarcoplasmic reticulum
plasma membrane
cell wall
protein histidine kinase complex
extracellular region
chloroplast
SCF ubiquitin ligase complex
mitochondrion
nucleolus
DNA-directed RNA polymerase I
1,3-beta-glucan synthase complex
translation initiation factor
endoplasmic reticulum membrane
protein serine/threonine…
endosome
plant-type cell wall
DNA-directed RNA polymerase I
Smc5-Smc6 complex
mitochondrial inner membrane
translation release factor complex
anchored to plasma membrane
Nup107-160 complex
respiratory chain
proton-transporting V-type ATPase
RNA-induced silencing complex
viral nucleocapsid
cell
intracellular membrane-bound
HAUS complex
protein acetyltransferase complex
mitochondrial outer membrane
large ribosomal subunit
ribonucleoprotein complex
membrane
apoplast
endoplasmic reticulum
trans-Golgi network
Biological Process
Molecular Function
Cellular component

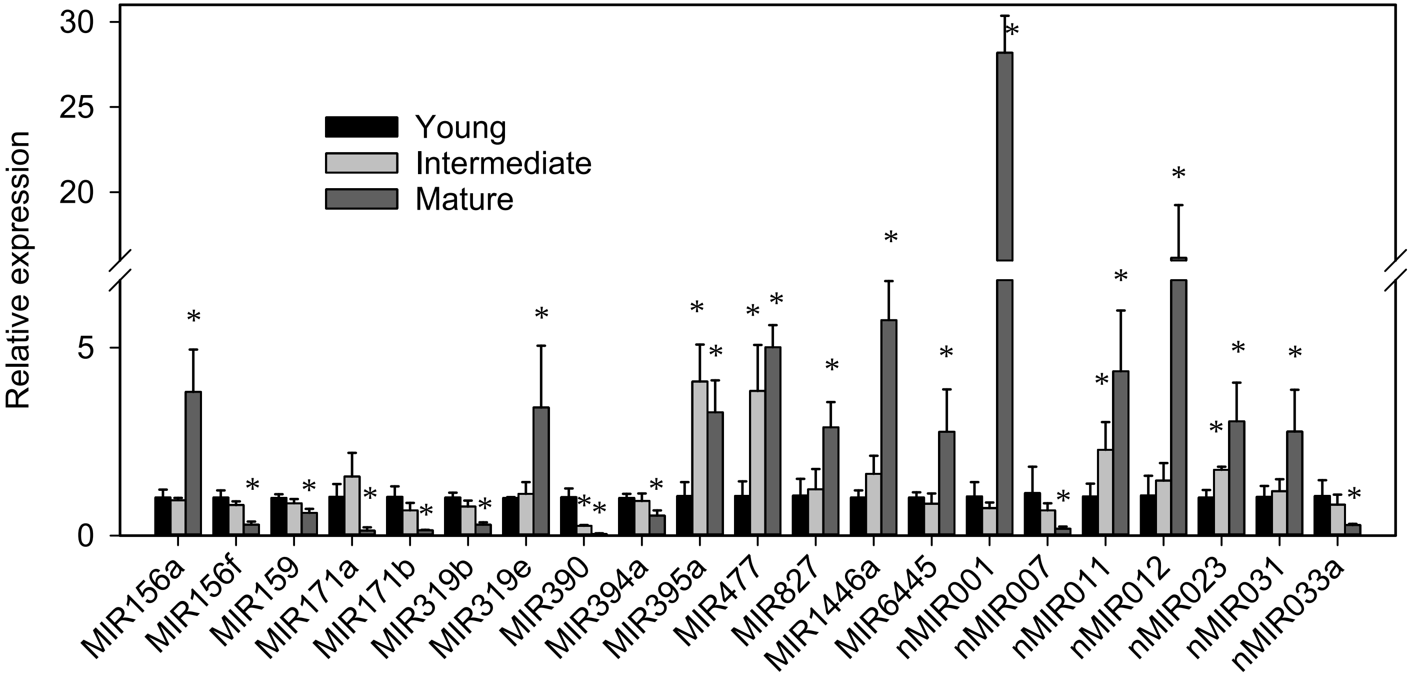

Relative expression
30
25
20
5
0
Young
Intermediate
Mature
MIR156a
MIR156f
MIR159
MIR171a
MIR171b
MIR319b
MIR319e
MIR390
MIR394a
MIR395a
MIR477
MIR827
MIR1446a
MIR6445
nMIR001
nMIR007
nMIR011
nMIR012
nMIR023
nMIR031
nMIR033a
